# The RNA Helicase DHX34 Activates NMD by Promoting a Transition from the Surveillance to the Decay-Inducing Complex

**DOI:** 10.1016/j.celrep.2014.08.020

**Published:** 2014-09-15

**Authors:** Nele Hug, Javier F. Cáceres

**Affiliations:** 1 MRC Human Genetics Unit, Institute of Genetics and Molecular Medicine, University of Edinburgh, Western General Hospital, Edinburgh EH4 2XU, UK

## Abstract

Nonsense-mediated decay (NMD) is a surveillance mechanism that degrades aberrant mRNAs. A complex comprising SMG1, UPF1, and the translation termination factors eRF1 and eRF3 (SURF) is assembled in the vicinity of a premature termination codon. Subsequently, an interaction with UPF2, UPF3b, and the exon junction complex induces the formation of the decay-inducing complex (DECID) and triggers NMD. We previously identified the RNA helicase DHX34 as an NMD factor in *C. elegans* and in vertebrates. Here, we investigate the mechanism by which DHX34 activates NMD in human cells. We show that DHX34 is recruited to the SURF complex via its preferential interaction with hypophosphorylated UPF1. A series of molecular transitions induced by DHX34 include enhanced recruitment of UPF2, increased UPF1 phosphorylation, and dissociation of eRF3 from UPF1. Thus, DHX34 promotes mRNP remodeling and triggers the conversion from the SURF complex to the DECID complex resulting in NMD activation.

## Highlights

• DHX34 interacts with Nonsense-mediated decay factors and the mRNA decay factory
• Hypophosphorylated UPF1 recruits DHX34 to the SURF complex
• DHX34 promotes the recruitment of UPF2, UPF1 phosphorylation, and eRF3 release
• DHX34 remodels mRNPs and promotes the transition from the SURF to DECID complex

## Graphical Abstract

**Figure:**
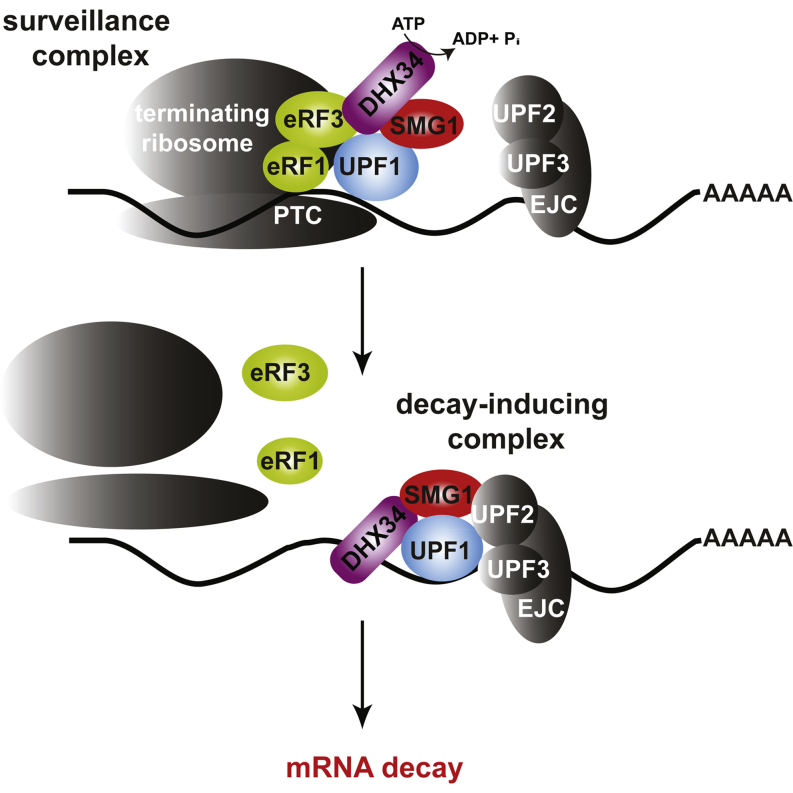

## Teaser

Nonsense-mediated mRNA decay (NMD) is an mRNA quality-control mechanism that acts to degrade mRNAs harboring premature termination codons (PTCs), preventing the accumulation of truncated proteins. Hug and Cáceres now find that the DExH/D box RNA helicase DHX34 interacts with several components of the core NMD machinery. It promotes a series of molecular transitions that are a hallmark of conversion from the PTC recognition complex to the mRNA degradation complex resulting in NMD activation.

## Introduction

Nonsense-mediated mRNA decay (NMD) is a surveillance pathway that eliminates mRNAs that contain premature termination codons (PTCs) preventing the synthesis of truncated proteins (reviewed by Chang et al., 2007; Isken and Maquat, 2008; Kervestin and Jacobson, 2012). This quality-control mechanism has a central role in the control of gene expression because it also targets for degradation a subset of naturally occurring transcripts (Schweingruber et al., 2013). Regulation by NMD can also have an important role in the modulation of the phenotypic outcome of many inherited genetic disorders that arise as consequence of a frameshift or mutations that generate premature termination codons (Bhuvanagiri et al., 2010). The basal NMD machinery consist of seven conserved genes that were initially identified by genetic screens in the nematode *C. elegans* and were later shown to be involved in this pathway in other organisms, including insects, plants, and vertebrates. In humans, these are SMG1, UPF1, UPF2, UPF3, SMG5, SMG6, and SMG7 (Nicholson et al., 2010). The NMD response is tightly controlled by a negative-feedback regulatory mechanism that controls the levels of core NMD factors in response to environmental stresses (Huang et al., 2011; Yepiskoposyan et al., 2011).

In vertebrates, pre-mRNA splicing is coupled to NMD via the exon junction complex (EJC), a multiprotein complex deposited on exon junctions following pre-mRNA splicing that recruits factors involved in NMD, mRNA export, and mRNA localization (Le Hir et al., 2001; Lykke-Andersen et al., 2001). The ribosome terminates prematurely at a PTC leaving downstream one or more EJC complexes, which are not removed from the mRNA and subsequently will recruit the NMD machinery. The essential splicing factor CWC22 interacts with the EJC factor eIFA3 and directly activates NMD by coupling splicing to EJC deposition (Alexandrov et al., 2012; Barbosa et al., 2012; Steckelberg et al., 2012).

The central NMD factor is the ATP-dependent RNA helicase, UPF1, known as SMG-2 in nematodes, whose phosphorylation is critical to trigger the NMD response. The SMG1c complex comprising the protein kinase SMG1, a phosphoinositide 3-kinase (PI3K)-like kinase, and the SMG8 and SMG9 subunits phosphorylates UPF1 at multiple [S/T]Q motifs at its C-terminal domain (reviewed by Yamashita, 2013). UPF1 is recruited to a PTC via interactions with the translation release factors, eRF1 and eRF3, leading to the assembly of a complex termed SURF that comprises SMG1 and UPF1, as well as eRF1 and eRF3. The SURF complex interacts with a downstream EJC via interactions with UPF2 and UPF3 proteins to form the decay-inducing complex (DECID) that triggers UPF1 phosphorylation and the dissociation of eRF1, 3 (Kashima et al., 2006). Subsequently, phosphorylated UPF1 recruits additional NMD factors (SMG5, SMG6, and SMG7) via their 14-3-3 domains that promote direct interactions with phosphoresidues in UPF1 and further rearrangements of this complex lead to mRNA degradation. Whereas SMG5 and SMG7 bound to UPF1 provide a link to mRNA decay (Jonas et al., 2013; Loh et al., 2013), SMG6 exhibits an endonuclease activity that is required for NMD in *Drosophila* and in vertebrates (Eberle et al., 2009; Huntzinger et al., 2008; Wittkopp et al., 2009). Importantly, the precise mechanism that controls the transition from the SURF to the DECID complex is not fully understood.

Recently, additional *trans*-acting factors involved in the NMD pathway have been uncovered, highlighting the complexity of this surveillance mechanism. A proteomic approach searching for novel SMG1 interactors led to the identification of two adenosine triphosphatases (AAA+) family proteins RuvB-like 1 and 2 (RUVBL1, RUVBL2) that promote SMG1 abundance and NMD complex formation in a process involving their ATPase activities (Izumi et al., 2010). A genome-wide RNAi screen in *C. elegans* resulted in the identification of two additional NMD factors, termed *smgl-1* and *smgl-2*, that, unlike *smg-1-7*, are essential for viability (Longman et al., 2007). These genes are highly conserved throughout evolution and their human homologs, NBAS (Neuroblastoma amplified sequence) and DHX34, function in the NMD pathway in human cells and also in zebrafish (Anastasaki et al., 2011; Longman et al., 2007).

Here, we provide a thorough molecular characterization of the role of DHX34 in the NMD pathway in human cells. We show that DHX34 is an RNA binding protein that interacts with several NMD factors, including UPF1 and its associated kinase, SMG1, as well as with proteins required for RNA degradation. We also show that recruitment of DHX34 to the SURF complex is required to promote the interaction of UPF1 with UPF2, and this results in UPF1 phosphorylation and release of eRF3. Altogether, these data suggest that recruitment of DHX34 to the SURF complex promotes UPF1 phosphorylation and triggers the conversion from the SURF complex into the DECID complex resulting in NMD progression and mRNA degradation.

## Results

### DHX34 Is an RNA Binding Protein

**Figure 1:**
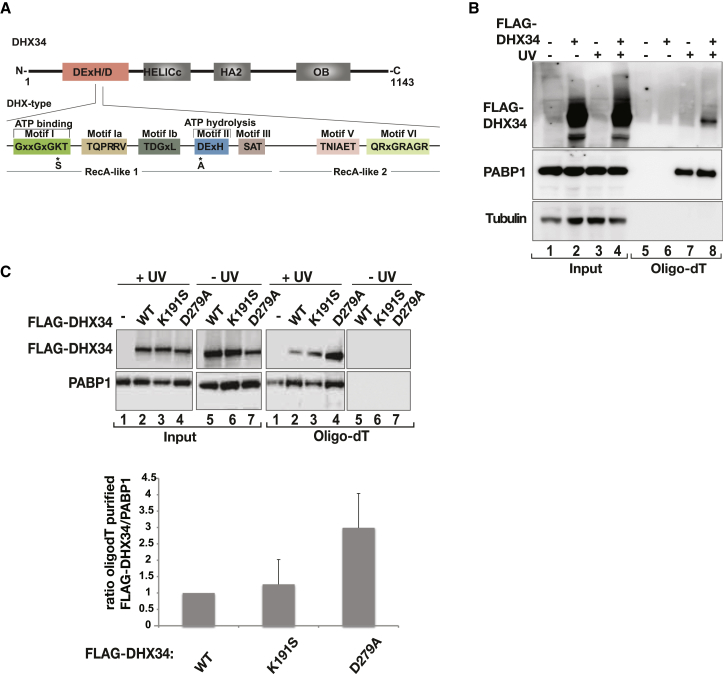
DHX34 Is an RNA Binding Protein (A) Cartoon depicting the functional domains of DHX34. The lower panel depicts the motifs present in the DExH/D box helicase domain, as well as two mutations in individual domains responsible for ATP binding and ATP hydrolysis, respectively (shown with asterisks). (B) HEK293T cells transiently transfected with FLAG-DHX34 were subjected to in situ UV crosslinking, followed by purification of mRNP complexes using Oligo dT chromatography under denaturing conditions. Lanes 1–4 (0.7% Input) contain extracts prior to Oligo dT selection, whereas lanes 5–8 (20% Oligo dT selected) contain purified mRNPs eluted from Oligo dT cellulose. Anti-FLAG was used to visualize the presence of DHX34 bound to purified mRNPs in western blot assays. An antibody that reacts with poly (A) binding protein (PABP1) was used as a purification control, whereas antitubulin served as a negative control. (C) The RNA binding ability of the DHX34 mutant proteins depicted in (A) was assayed by an mRNA capture assay, as described in (B). The lower panel shows the ratio of oligo dT cellulose purified FLAG-DHX34 protein over PABP1 from at least two independent experiments.

Human DHX34 *(DEAH box protein 34*) belongs to the DExH/D box family of proteins that use ATP hydrolysis to promote directly or indirectly RNA-RNA unwinding, RNA-protein dissociation, and protein-protein interactions (Jankowsky, 2011; Linder and Jankowsky, 2011). It contains four domains, including one helicase domain (DExH/D) and three not well-characterized domains (HELICc, HA2 and a C-terminal OB [Oligonucleotide/oligosaccharide binding] fold), which are often found associated with the helicase domain in this group of proteins (Figure 1A). The highly conserved DExH/D helicase core consists of two recombinase A (RecA)-like helicase domains (domains 1 and 2), comprising well-conserved sequence motifs (I–VI) that harbor the binding sites for ATP, the ATPase activity as well as sequences responsible for RNA binding. The motif II contains the DExH/D sequence, which gives the name to this protein family (Figure 1A). First, we tested whether DHX34 directly binds to mRNA. For this, we used an mRNA capture assay, which consists of in situ UV crosslinking and denaturing oligo dT selection to purify messenger ribonucleoprotein particles (mRNPs) from HEK293T cells, as previously described (Piñol-Roma and Dreyfuss, 1992; Sanford et al., 2005). Affinity selection of mRNPs by oligo dT cellulose clearly demonstrated that DHX34 is bound to mRNA (Figure 1B). In order to determine the contribution of ATP binding and/or hydrolysis, we mutated the lysine (K) residue in the Walker A motif GXXXXGK(T/S) (K191S in motif I), which is crucial for nucleotide binding, as well as the aspartate (D) residue in the Walker B motif (D279A in motif II) that is required for ATP hydrolysis (Hanson and Whiteheart, 2005) (Figure 1A) and analyzed the ability of these DHX34 mutant proteins to bind to RNA. Neither of the mutations disrupted RNA binding (Figure 1C) despite being highly overexpressed (Figure S1A). We observed that the K191S mutation that abrogates ATP binding showed similar mRNA binding compared to the wild-type protein. By contrast, the point mutation in the conserved aspartate residue of motif II (D279A), which abrogates ATP hydrolysis, displayed increased binding to mRNA. We also confirmed the binding of endogenous DHX34 protein by performing immunoprecipitations of UV-crosslinked RNA-protein complexes (Figure S1B). Altogether, these results demonstrate that DHX34 binds RNA directly and that a defect in ATP hydrolysis results in increased mRNA association suggesting that this might be required for the release of DHX34 from mRNA (Figure 1C), as has been seen in other instances (Henn et al., 2010; Liu et al., 2008).

### DHX34 Interacts with NMD Effectors

**Figure 2:**
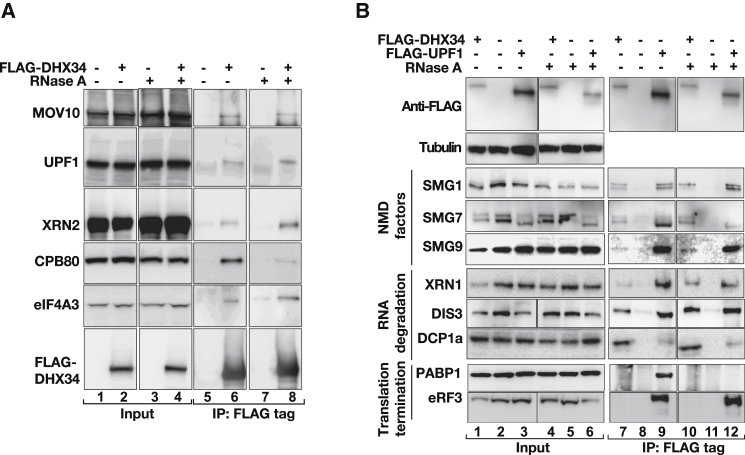
DHX34 Interacts with NMD Effectors (A) Cells were transiently transfected with FLAG-DHX34. Anti-FLAG IPs were performed in the presence or absence of RNase A. Inputs (1%) and anti-FLAG IPs (25%) were subjected to western analysis and probed for the indicated proteins. (B) Immunoprecipitation of transiently transfected FLAG-DHX34 and FLAG-UPF1 from HEK293T cells in the absence or presence of RNase A. Inputs (0.5%) and anti-FLAG IPs (20%) were subjected to western analysis using the indicated antibodies.

In order to dissect the role of DHX34 in the NMD pathway, we used a combination of immunoprecipitation (IP)-mass spectrometry with a candidate approach in which we tested for interactions with known NMD components. First, we immunopurified endogenous DHX34 from HEK293T cells and determined the associated proteins using mass spectrometry. This analysis was carried out in the absence of RNases in order to identify the composition of DHX34 mRNPs. For all subsequent validation experiments, we treated the lysates with RNases (see below). This experiment resulted in the identification of several RNA binding proteins, including proteins linked to the NMD pathway, and many other RNA processing events (Figures S2A and Table S1). From this list, we validated five interactors that have been previously described to function in NMD: the core factor UPF1, the RNA helicase MOV10 (Gregersen et al., 2014), the EJC component, eIF4A3, the mRNA Cap-binding protein, CPB80, and the 5′ to 3′ exonuclease XRN2. We transiently expressed FLAG-tagged DHX34 and following anti-FLAG Immunoprecipitation in the presence or absence of RNases, we analyzed the corresponding proteins by western blot analysis. A control for the activity of RNase in the degradation of cellular RNAs is provided (Figure S2E). We observed that DHX34 interacts in an RNA-independent manner with UPF1, MOV10, XRN2, and eIF4A3, whereas its interaction with CBP80 is RNA dependent (Figure 2A). We confirmed the interaction of DHX34 with UPF1 by showing the copurification of endogenous DHX34 protein with epitope-tagged FLAG-UPF1 in an RNA-independent manner (Figure S2B) as well as by the coimmunoprecipitation of endogenous DHX34 and UPF1 proteins (Figure S2C). To better understand the function of DHX34 in NMD, we tested the interaction of DHX34 with known NMD factors and RNA degradation factors, in the presence or absence of RNases. We found that FLAG-tagged DHX34 coimmunoprecipitated the NMD factors SMG1, SMG7, SMG9, SMG6, and UPF3a in an RNA-independent manner (Figures 2B and S2D). We could also detect RNA-dependent interactions of DHX34 with UPF3b and the EJC component, MLN51 (Figure S2D). No interaction could be detected with PABP1 or with the eukaryotic release factor eRF3 (Figure 2B), nor with the NMD core factor UPF2 (Figure S2D). By contrast, we found a positive interaction for these factors with FLAG-UPF1, as previously reported (reviewed by Mühlemann et al., 2008) (Figures 2B and S2B). Under these conditions PABP1 interacts with UPF1 only in the presence of RNA. We also detected interactions of DHX34 with RNA degradation factors such as the exonuclease XRN1, the exosome component DIS3, and the decapping enzyme DCP1, all of them independently of the presence of RNA (Figure 2B). In summary, a combination of an IP-mass spectrometry approach with a candidate analysis revealed that DHX34 interacts with several components of the NMD pathway, including core NMD factors as well as RNA degrading components (summarized in Figure S2F).

### DHX34 Interacts with UPF1 Directly and Preferentially Associates with SURF Complexes

**Figure 3:**
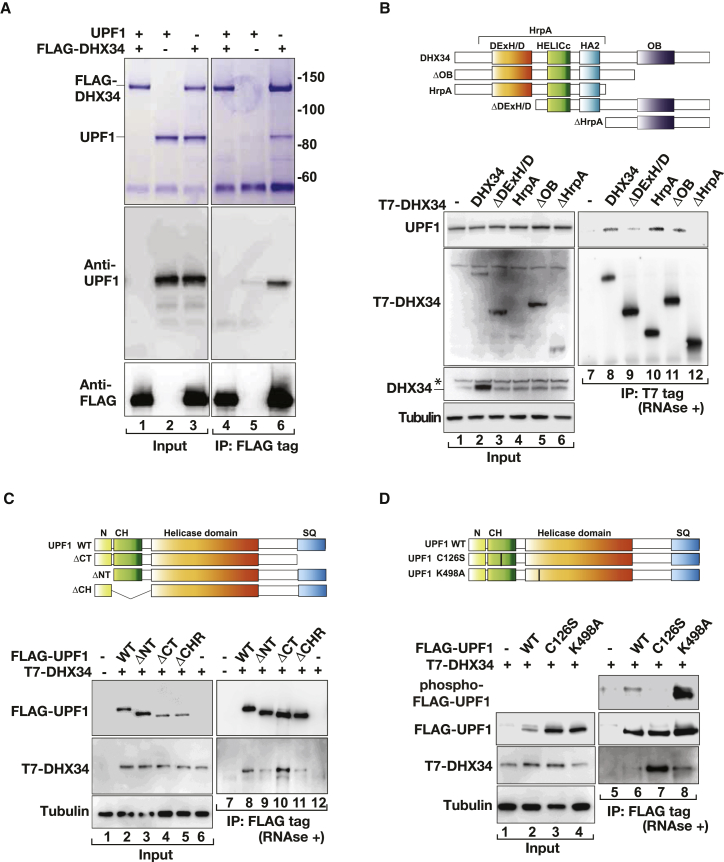
DHX34 Interacts with UPF1 and Preferentially Associates with SURF Complexes (A) Interaction between purified FLAG-DHX34 and recombinant UPF1 proteins, which were mixed in a 1:1 ratio and pulled down using anti-FLAG beads. FLAG-DHX34 was purified from HEK293T cells under stringent conditions, whereas recombinant UPF1 protein was purified from bacteria. Inputs (5%) and pull-down fractions (20%) were analyzed after SDS-PAGE by Coomassie staining or western blots, with the indicated antibodies. (B) The upper panel shows the domain structure organization of DHX34, already described on Figure 1A. Immunoprecipitation of transiently transfected T7-DHX34 (wild-type or deletion mutants) from HEK293T cells was performed in the presence of RNase A. Inputs (0.5%) and anti-FLAG IPs (20%) were subjected to western blot analysis using the indicated antibodies. The level of overexpression of wild-type T7-DHX34 was determined by comparison with levels of endogenous DHX34 protein, which was probed with a specific antibody (input panel). The lower band corresponds to DHX34, whereas an asterisk above DHX34 indicates an unspecific band. (C) The upper panel shows the domain organization of UPF1 and the corresponding deletion mutants. FLAG IPs from cells transiently cotransfected with FLAG-UPF1 (wild-type or deletion mutants) and T7-DHX34 were performed in the presence of RNase A. Inputs (0.5%) and anti-FLAG IPs (20%) were subjected to western blot analysis using the indicated antibodies. (D) DHX34 binds preferentially nonphosphorylated UPF1. The upper panel shows the domain structure of UPF1, with the point mutations introduced in the ATP binding domain of UPF1 (K498A) or in the UPF1 C126S mutant, indicated with a vertical bar. FLAG-UPF1 (wild-type or mutants thereof) was coexpressed with T7-DHX34 in HEK293T cells. Input (0.5%) and anti-FLAG IPs (20%) were subjected to western blot analysis for the indicated proteins. To detect phosphorylated UPF1 (phospho-FLAG-UPF1), anti-FLAG IPs were probed with a phospho-(Ser/Thr) ATM/ATR substrate antibody.

Next, we probed for a direct interaction of UPF1 and DHX34 by coincubating FLAG-DHX34 purified from HEK293T cells with recombinant UPF1 protein. We found that FLAG-DHX34 was able to pull-down recombinant UPF1 protein (Figure 3A). This strongly suggests that DHX34 and UPF1 interact directly, even though we cannot formally exclude that this interaction could be mediated through the presence of a copurified bridging protein. FLAG-DHX34 interaction with UPF1 was still observed upon RNase treatment (Figure S3A); however, we could not observe any binding of UPF1 to the unrelated protein FLAG-KAT8 under the same conditions (Figure S3A). Furthermore, we noticed that, whereas T7-tagged DHX34 binds very strongly to purified full-length FLAG-UPF1, it has only a very weak affinity for UPF2 and UPF3b (Figure S3B, compare lanes 9, 11, and 14). We next analyzed the functional domains in DHX34 that are required for its interaction with endogenous UPF1 by overexpressing T7-tagged DHX34 (wild-type and deletion mutant proteins). We observed that deletion of the entire HrpA (ΔHrpA) region abolished binding to UPF1, whereas ΔDExH/D displayed a reduced interaction. All the remaining deletion mutants could still interact with UPF1 (Figure 3B). This was recapitulated in vitro by coincubation of purified T7-DHX34 (wild-type and mutant proteins) with recombinant UPF1 proteins, which again showed a reduced interaction of T7-DHX34ΔHrpA with UPF1 (Figure S3C, compare lanes 13 and 16). We next used immunoprecipitation assays of HEK293T cells coexpressing FLAG-UPF1 (wild-type and deletion mutants) and T7-tagged DHX34 to delineate the domains of UPF1 that are required for its interaction with DHX34. UPF1 consists of an N-terminal cysteine-histidine domain (CH) regulatory domain, which is bound by UPF2 (Cheng et al., 2007; Clerici et al., 2009; Kadlec et al., 2006), a central helicase domain and a C-terminal tail that contains several S/T Q (SQ) motifs that are phosphorylated by SMG1 (Ohnishi et al., 2003). DHX34 showed a slightly reduced interaction with the deletion mutants lacking the N-terminal tail (ΔN) and the globular CH domain (ΔCH) (Figure 3C, compare lanes 8, 9, and 11). Most interestingly, the deletion lacking the C terminus (ΔCT) comprising the SMG1-phosphorylation sites showed an increased association with DHX34 compared to the wild-type protein, which is suggestive of DHX34 binding to nonphosphorylated UPF1 (Figure 3C, compare lanes 8 and 10). UPF1 (wild-type and deletion mutants) were expressed at similar levels to the endogenous protein (Figure S3D). The observation that DHX34 preferentially binds to UPF1 lacking its phosphorylated C terminus raised the question whether DHX34 binds to hypophosphorylated UPF1. Because UPF1 phosphorylation is a later step in NMD activation (Kashima et al., 2006), we wanted to investigate the temporal sequence during which DHX34 is recruited to UPF1-containing NMD complexes. We took advantage of a series of UPF1 mutant proteins that affect distinct functions of this protein. First, we observed that the FLAG-tagged K498A substitution mutant, which affects an ATP binding residue and abolishes the ATPase activity of UPF1 (Cheng et al., 2007) and is hyperphosphorylated displayed similar binding to T7-tagged DHX34 as the wild-type FLAG-UPF1 protein (Figure 3D, lanes 6 and 8). By contrast, the C126S mutation in UPF1 that prevents its interaction with UPF2 and consequently freezes the surveillance complex (Kashima et al., 2006; Weng et al., 1996) displayed a strong enrichment of DHX34 binding compared to wild-type UPF1 (Figure 3A, compare lanes 6 and 7). This suggested that DHX34 is preferentially recruited to the SURF complex, where UPF1 is hypophoshorylated. In agreement with this, a different UPF1 mutant protein (G495R/G497E), which is hyperphosphorylated (Figure S3E, lanes 5–8) probably due to a helicase defect similar to the K498A substitution (Page et al., 1999), showed even reduced DHX34 association (Figure S3E). Altogether, these experiments strongly suggest that DHX34 is preferentially recruited to the SURF complex.

### DHX34 Promotes UPF1 Phosphorylation

**Figure 4:**
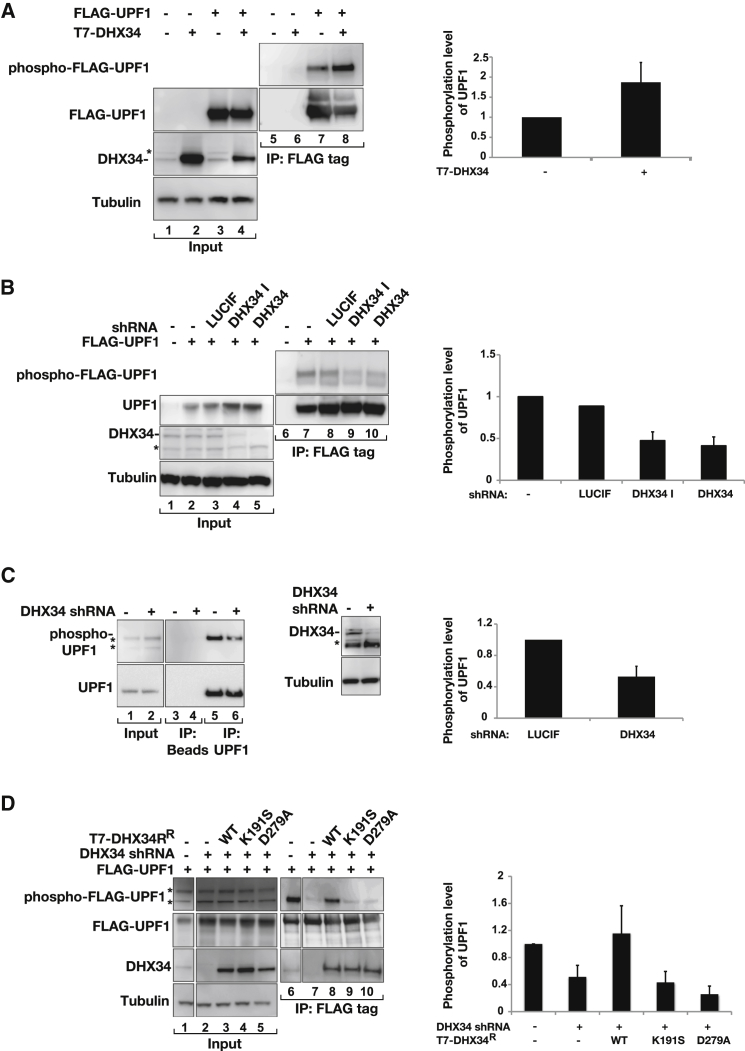
DHX34 Promotes UPF1 Phosphorylation (A) HEK293T cells were transiently transfected with FLAG-UPF1 in the absence or presence of cotransfected T7-DHX34. Inputs (0.5%) and anti-FLAG IPs (20%) were probed with the indicated antibodies. Phosphorylated UPF1 was detected with a phospho-(Ser/Thr) ATM/ATR substrate antibody. The Phospho-FLAG-UPF1 signal was normalized to the levels of UPF1 recovered in the IP. A quantification of relative levels and SDs of the western blot signals from three independent experiments are shown (right panel). (B) HEK293T cells depleted of DHX34 with a specific shRNA (with empty vector plasmids or an shRNA targeting Luciferase (LUCIF) used as negative controls) for 4 days were then transfected with FLAG-UPF1 or an empty vector control and immunoprecipitated 3 days later. Phosphorylation levels of UPF1 were determined as described above. (C) Detection of phospho- UPF1 was as described above despite that cells were transfected with DHX34 shRNA or an shRNA targeting Luciferase (−) and endogenous UPF1 was immunopurified 5 days later. Inputs (0.5%) and IPs (20%) were probed with the indicated antibodies. Asterisks indicate phosphorylated proteins (distinct from UPF1) recognized with phospho-(Ser/Thr) ATM/ATR substrate antibodies in the input samples. Depletion of DHX34 is shown on the right, with the top band corresponding to DHX34, whereas the asterisks below indicate unspecific bands. Quantification of the western blot signal and SDs from two independent experiments are shown (right panel). (D) HEK293T cells depleted of DHX34 with specific shRNA or transfected with an shRNA targeting Luciferase (−) were cotransfected with FLAG-UPF1 and shRNA-resistant (^R^) wild-type T7-DHX34 or ATPase-deficient version (K191S or D279A). Phosphorylated UPF1 was detected as described above. Asterisks indicate phosphorylated proteins (distinct from UPF1) that were also recognized with phospho-(Ser/Thr) ATM/ATR substrate antibodies in the input samples. A quantification of the western blot signal and SDs from three independent experiments are shown (right panel).

Because we detected interactions of DHX34 with UPF1 and with its protein kinase SMG1 and found that DHX34 binds preferentially to the SURF complex, we next tested whether DHX34 affects UPF1 phosphorylation. We immunoprecipitated FLAG-tagged UPF1 and analyzed its phosphorylation status in the presence or absence of DHX34 using a phospho-(Ser/Thr) ATM/ATR substrate antibody. We found that UPF1 phosphorylation increased upon overexpression of T7-DHX34 (Figure 4A, compare lanes 7 and 8). Conversely, small hairpin RNA (shRNA)-mediated depletion of DHX34 resulted in a marked decrease in UPF1 phosphorylation (Figure 4B). The effect on UPF1 phosphorylation correlated well with the level of DHX34 overexpression (Figures S4A and S4B), and a reduction in the phosphorylation of endogenous UPF1 protein was also observed upon DHX34 depletion (Figure 4C). Importantly, the levels of UPF1 phosphorylation were rescued upon the expression of an shRNA-resistant wild-type DHX34 construct, but not by the ATPase-deficient DHX34 mutants (K191S or D279A), which affect ATP binding or ATP hydrolysis, respectively (Figure 4D). Next, we investigated whether DHX34 has a direct effect on UPF1 phosphorylation. For this, we performed in vitro kinase assays with purified recombinant SMG1 protein and monitored its kinase activity on SMG1 autophosphorylation (Morita et al., 2007) as well as on UPF1, in the presence or absence of added DHX34. We noticed that the addition of purified DHX34 had no effect on the SMG1 kinase activity on neither of the substrates (Figure S4C). These experiments suggest that the effect of DHX34 on UPF1 phosphorylation is not direct and could reflect that DHX34 activates a molecular event that indirectly triggers UPF1 phosphorylation (see below).

### DHX34 Promotes Interaction of UPF1 with UPF2 and the EJC Complex

**Figure 5:**
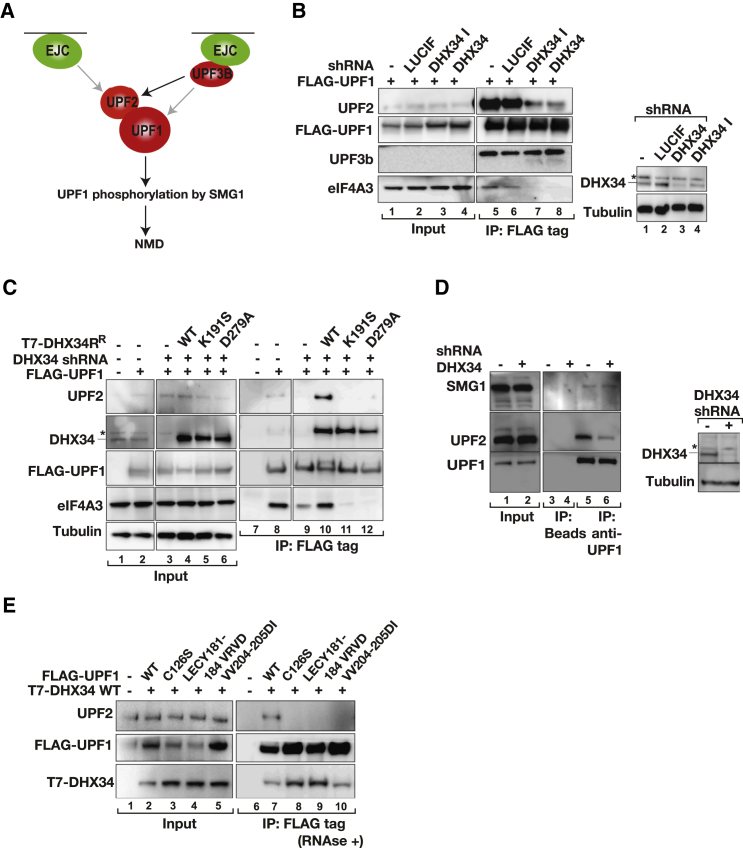
DHX34 Promotes the Binding of UPF2 to UPF1 (A) Cartoon depicting the different branches of the NMD pathway. (B) HEK293T cells were cotransfected with the indicated shRNA constructs and FLAG-UPF1 and subjected to anti-FLAG IPs. Input (0.5%) and anti-FLAG IPs (20%) were analyzed by western blotting with the indicated antibodies. The right panel shows the depletion of DHX34 for the input samples. The lower band represents DHX34. The asterisk above DHX34 indicates an unspecific band. (C) HEK293T cells were transfected with an shRNA targeting DHX34 or with an empty vector control. After 4 days, cells were cotransfected with FLAG-UPF1 and an shRNA-resistant (^R^) T7-DHX34 (wild-type or the ATPase-deficient mutants K191S or D279A). Input (0.5%) and anti-FLAG IPs (20%) were analyzed by western blotting with the indicated antibodies. (D) Endogenous UPF1 protein complexes were immunoprecipitated from HEK293T cells depleted of DHX34. The Immunoprecipitates were probed for proteins indicated on the left. (E) Inputs (0.5%) and anti-FLAG IPs (20%) of cells coexpressing FLAG-UPF1 (wild-type, or the C126S, LECY181-184VRVD, or VV204-205 mutants) and T7-DHX34 were analyzed for the indicated proteins.

The NMD pathway can be activated via different branches that differ in their dependence on the NMD factors UPF2, UPF3b, and EJC components (Chan et al., 2007; Gehring et al., 2005; Ivanov et al., 2008); however, they all require UPF1 to activate NMD (Figure 5A). Because DHX34 preferentially associates with SURF complexes and promotes UPF1 phosphorylation, we tested the formation of the DECID complex in the absence of DHX34. We observed a reduced interaction of UPF1 with UPF2 and with eIF4A3 in cells depleted of DHX34 (Figure 5B; compare lanes 5 and 6 with lanes 7 and 8). By contrast, no reduced interaction was observed for SMG1, SMG7, XRN2, whereas a slightly increased binding of SMG9 was observed (Figure S5A). Whereas expression of an shRNA-resistant wild-type DHX34 restored the interaction of UPF1 with UPF2 and with eIF4A3 (Figure 5C, lane 10), the expression of the ATPase-deficient DHX34 mutants (K191S or D279A) failed to achieve this (Figure 5C, lanes 11 and 12). Importantly, we noticed that upon depletion of DHX34 the association of endogenous UPF2 with UPF1 was drastically reduced (Figure 5D). In order to delineate the branch of the NMD pathway that is affected by DHX34, we took advantage of a series of UPF1 mutant proteins (C126S, LECY181-184VRVD, and VV204-205DI), all of which have lost UPF2 binding (Figure 5E). An important functional distinction is that, although the first two UPF1 mutants are inactive in NMD, the VV204-205DI mutant can use the UPF3b alternative branch of the NMD pathway (Ivanov et al., 2008). We found that DHX34 preferentially binds UPF1 C126S and LECY181-184VRVD mutant proteins (Figure 5E). However, we could not observe an increased binding of DHX34 to the VV204-205DI mutant (Figure 5E, compare lanes 7–10), suggesting that DHX34 does not affect the UPF3b-dependent branch of NMD. This is supported by the fact that DHX34 does not influence UPF1 binding to UPF3b (Figure 5B). To further dissect the involvement of DHX34 in different NMD branches, we performed gene expression analysis using Agilent microarrays of HeLa cells individually depleted of DHX34, UPF1, UPF2, or UPF3b. We clearly observed that DHX34 and the core NMD factors UPF1, UPF2, and UPF3b show a significant positive correlation in the regulation of endogenous RNA targets (p value for all comparisons <2.2 × 10^−16^) (Figure S5B). Next, we focused on genes upregulated upon depletion of UPF1 and DHX34 (n = 800), UPF2 (n = 896), or UPF3b (n = 310). We defined these as bona fide NMD targets. From this analysis, we conclude that DHX34 coregulates a significant proportion of RNA targets with UPF2 and UPF1 (n = 277) and with UPF3b and UPF1 (n = 166) (Figure S5C). Together, with the biochemical evidence presented above, this would suggest that DHX34 is not involved in an alternative branch of the NMD pathway; rather, it seems to activate the canonical NMD pathway.

### DHX34 Triggers the Conversion of the SURF to the DECID Complex

**Figure 6:**
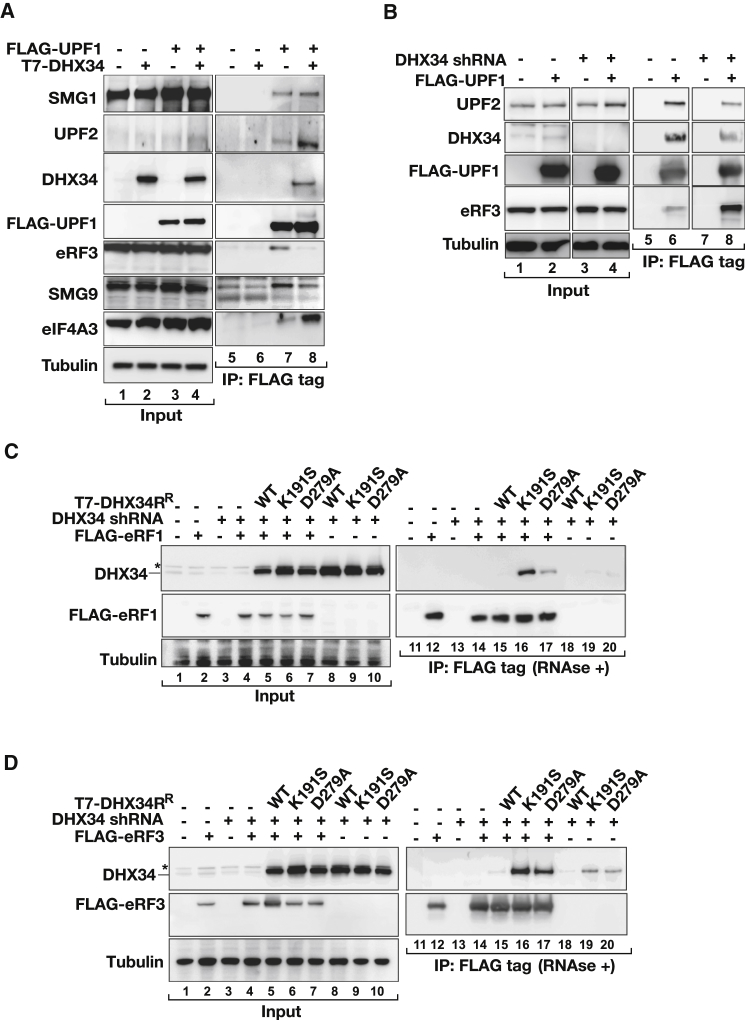
DHX34 Promotes eRF3 Release from the SURF Complex (A) FLAG-UPF1 was transiently coexpressed with T7-DHX34 in HEK293T cells. Inputs (0.5%) and anti-FLAG IPs (20%) were analyzed with the indicated antibodies. (B) HEK293T cells were transiently transfected with FLAG-UPF1 and with an shRNA targeting DHX34 or with an empty vector control. Inputs (0.5%) and anti-FLAG (20%) IPs were analyzed by western blotting for the indicated proteins. (C and D) Cells were transfected with an shRNA targeting DHX34 or with an empty vector control. After 4 days cells were cotransfected with FLAG-eRF1 (C) or FLAG-eRF3 (D) and shRNA-resistant (^R^) T7-DHX34 (wild-type or the ATPase-deficient mutants K191S and D279A). Anti-FLAG immunoprecipitations were performed 2 days later and analyzed for the presence of DHX34. Input (0.5%) and anti-FLAG IPs (20%) were analyzed by western blotting with the indicated antibodies.

Results presented so far are compatible with a role for DHX34 in the active remodeling of the SURF complex promoting its transition to the DECID complex. This is supported by the finding that overexpression of DHX34 results in the release of eRF3 from UPF1 complexes, which is a hallmark of this transition (Figure 6A, lanes 7 and 8). By contrast, the interaction of UPF1 with SMG1 was not impaired (Figure 6A); however, a decreased recruitment of SMG9, a subunit of the SMG1c complex, was also observed (Figure 6A) as well as the DHX34-dependent recruitment of UPF2 and eIF4A3 (Figures 5 and 6A). Interestingly, the interaction of UPF1 with eIF3A or CBP80 was not affected (Figure S6A), strongly suggesting that DHX34 does not activate NMD by either promoting translational repression (via a phospho-Upf1-eIF3 interaction) (Isken et al., 2008) or by stimulating the interaction of UPF1 with CBP80 (Hwang et al., 2010). Conversely, in the absence of DHX34 we observed an increased association of eRF3 with UPF1 (Figure 6B, compare lanes 6 and 8). We previously failed to detect an interaction of wild-type DHX34 with the translation release factor eRF3 (Figure 2B). We reasoned that if eRF3 release is triggered upon DHX34 ATP hydrolysis, we would be able to detect the DHX34-eRF3 interaction using the catalytically inactive mutants of DHX34. Thus, we depleted endogenous DHX34 and coexpressed shRNA-resistant T7-tagged DHX34 (wild-type and the ATPase mutants K191S and D279A) with FLAG-eRF1 (Figure 6C) or FLAG-eRF3 (Figure 6D). In agreement with previous results (Figure 2B), wild-type T7-DHX34, although expressed at a high level, failed to interact with eRF1 and eRF3. Interestingly, we could detect the two ATPase mutants of DHX34 associated with eRF1 (Figure 6C) and eRF3 (Figure 6D). By contrast, we could not find any differences in the binding of the two ATPase mutants to any other member of the SURF complex. For instance, we found that SMG1 coimmunoprecipitated with T7-tagged DHX34 (wild-type or mutants) to a similar extent (Figure S6B). Furthermore, DHX34 (wild-type and mutants) showed preferential binding to the C126S and LECY181-184VRVD UPF1 mutants that freeze the SURF complex (Figures S6C–S6E).

Altogether, these results suggest that DHX34 supports the transition from SURF to DECID resulting in UPF1 phosphorylation and release of the eRFs from the PTC resulting in targeting of the faulty mRNA for degradation. In summary, this work provides a mechanistic characterization of the role of the RNA helicase DHX34 in mRNP remodeling leading to activation of NMD.

## Discussion

We previously found that DHX34 acts in concert with core NMD factors to coregulate a significant number of endogenous RNA targets in *C. elegans*, zebrafish, and human cells (Anastasaki et al., 2011; Longman et al., 2007, 2013). Here, we provide a mechanistic analysis of the role of DHX34 in the NMD pathway in human cells. A combination of a mass spectrometry interactome with a gene candidate approach revealed interactions of DHX34 with mRNA degradation factors, as well as with the central NMD factor UPF1 and its kinase SMG1 (Figures 2 and S2). Despite being central for the NMD process, there is limited information on how the protein kinase SMG1 is activated and how the transition from the SURF to the DECID complex is achieved. The SMG8 and SMG9 subunits recruit SMG1 to the SURF complex and allosterically inhibit the kinase activity of SMG1 (Fernández et al., 2011; Yamashita et al., 2009). The subsequent interaction of the C terminus of SMG1 and UPF2 are believed to induce the dissociation of SMG8 and activation of SMG1 (Arias-Palomo et al., 2011; Yamashita et al., 2009).

DHX34 is recruited to the SURF complex via its preferential interaction with hypophosphorylated UPF1 and subsequently promotes UPF1 phosphorylation concomitantly with a disruption of the SURF complex (Figure 4). Importantly, we could not detect a direct role for DHX34 in the activation of SMG1 (Figure S4), suggesting a role of DHX34 in promoting a molecular event that indirectly results in the activation of the SMG1 kinase. It has been shown that UPF1 phosphorylation requires the interaction of components of the SURF complex with UPF2-UPF3-EJC downstream of a PTC (Kashima et al., 2006). Indeed, we were able to show that a major role for DHX34 is to promote the recruitment of UPF2 to the SURF complex together with the release of eRF3 (Figures 5 and 6). We propose that DHX34 acts by facilitating UPF1-UPF2 interactions that result in the remodeling of the SURF complex, leading to increased UPF1 phosphorylation as a consequence.

Although helicases were first described as ATPases that unwind polynucleotide duplexes, several members of the DExH/D box family function as RNPases that promote ATP-dependent RNP remodeling by removing proteins from the RNA in the absence of double-stranded RNA unwinding (Fairman et al., 2004; Schwer, 2001). For example, crystal structure analysis revealed that the DEAD box protein eIF4A3 binds RNA in an ATP-dependent fashion and serves as a platform for the nucleation of the EJC core factors (Andersen et al., 2006; Bono et al., 2006). Once loaded, this DEAD box RNA helicase can serve as a nucleation center to recruit additional proteins and establish a larger ribonucleoprotein complex. We cannot rule out that DHX34 helicase activity is additionally required to cause strand displacement of secondary RNA structure in the vicinity of a PTC. A role for an RNA helicase in remodeling protein-RNA complexes involved in NMD was recently shown, with the ATPase activity of UPF1 being required to promote the disassembly of mRNPs undergoing NMD (Franks et al., 2010). This function can be assisted by another RNA helicase MOV10, which assists UPF1-mediated mRNA degradation by resolving secondary structures and displacing proteins from 3′ UTRs (Gregersen et al., 2014).

**Figure 7:**
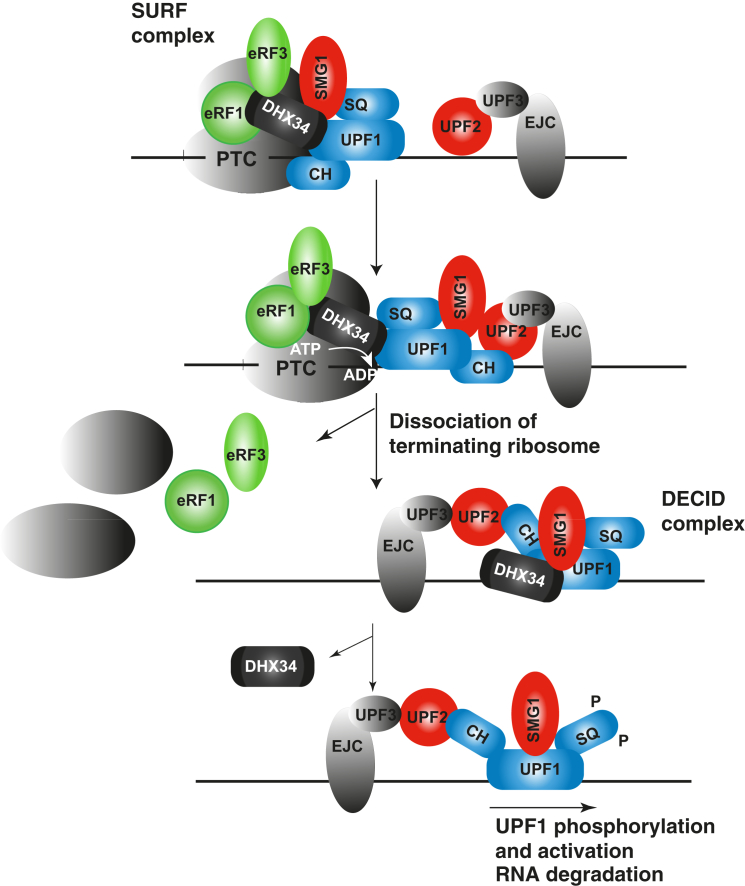
Model Depicting the Role of DHX34 in NMD Activation A translation termination event upstream of an exon junction complex leads to the recruitment of UPF1 and SMG1 by the eukaryotic translation release factors eRF1 and eRF3, forming the surveillance complex (SURF). At this stage, UPF1 activity is repressed by two distinct intramolecular interactions mediated by the N-terminal CH domain and the C-terminal SQ domain (Chakrabarti et al., 2011; Fiorini et al., 2013). During the assembly of the decay-inducing complex (DECID), the interaction of UPF1 with UPF2 induces a large conformational change in the regulatory CH domain of UPF1; however, complete activation is only achieved when repression of its SQ domain is relieved and the bound SMG1 kinase phosphorylates UPF1 on its SQ domain. This is accompanied by the displacement of the ribosome and the eRFs from the RNP complexes. This conversion is enhanced by DHX34, which associates with the SURF complex and promotes the remodeling of the SURF complex. DHX34 triggers the release of the release factors eRF1 and eRF3 in an ATP-hydrolysis-dependent manner and promotes the interaction of UPF1 with UPF2 and additional EJC proteins and induces the transition to the DECID complex that targets the RNA for decay.

We have clearly established that the ATPase activity of DHX34 is required to promote UPF2 recruitment to UPF1, to induce UPF1 phosphorylation and to dissociate the release factors eRF1/3. All these events are hallmarks of the transition from the SURF to the DECID complex (Figure 7). It is tempting to speculate that DHX34 function in NMD relies on its ability to promote the release of eRF1 and eRF3, preventing translation termination and allowing the NMD machinery to target faulty mRNAs for decay. In summary, results presented here highlight the complex nature of the molecular mechanisms that result in the activation of NMD. We propose that the DEAH (DExH/D) box RNA helicase DHX34 remodels NMD complexes in an ATP-driven manner causing a transition from SURF to the DECID complex that results in NMD activation.

## Experimental Procedures

### Transfection, Immunoprecipitation, and Western Blot Analysis

For shRNA transfections, cells grown in 6-well plates were transfected with 4 μg of plasmid shRNA pSuperpuro with Lipofectamine 2000 (Life Technologies) following manufacturer’s instructions and expanded 24 hr later into selective media containing 0.75 μg Puromycin (Sigma-Aldrich). For rescue experiments, cells were transfected 72 or 96 hr later with 10 μg of shRNA, together with 20 μg of pcDNA3 × FLAG-UPF1, as well as 20 μg of a plasmid expressing shRNA-resistant wild-type, mutant constructs, or empty vector controls, selected with medium containing puromycin and harvested for immunoprecipitation 48 or 72 hr later. For affinity purification, cells were transfected with 20 μg of T7-tagged or FLAG-tagged expression vectors. Cells were washed 48 hr later with 10 ml PBS and lysed in 2 ml IP buffer (10 mM Tris-HCl [pH 8], 150 mM NaCl, 1 mM EGTA, 1% NP-40, 0.2% Na-Deoxycholate, Complete Protease Inhibitor (Roche), Phospho STOP (Roche), 1 mM DTT). In some experiments, cell lysates were treated with 40–80 μg/ml RNase A per 1 ml extract. Protein complexes were analyzed by SDS-PAGE using 3%–8% Tris-Acetate or 5%–15% Tris-glycine gels (Life Technologies) followed by western blotting or mass spectrometry analysis. For immunoprecipitations of eRFs, cells were lysed in 20 mM Tris-HCl (pH 7.2), 150 mM KCl, 10 mM MgCl_2_, 0.5% NP-40, Complete Protease Inhibitor (Roche Diagnostics), Phospho STOP (Roche Diagnostics), and 1 mM DTT as previously described (Ivanov et al., 2008). Analysis of peptide samples by nLC-MS/MS and provision of MASCOT report was carried out by FingerPrints Proteomics Facility, University of Dundee. Interactions were considered as positive if they retained a significant score after subtracting polypeptides found with the preimmune serum control.

### In Vitro Pull-Down Assays

HEK293T cells were grown in 10 cm plates and transfected using Lipofectamine 2000 according to the manufacturer’s protocol. Two days after transfection, cells were lysed in IP buffer (10 mM Tris [pH 8], 150 mM NaCl, 1 mM EGTA, 1% NP-40, 0.2% Na-Deoxycholate, Complete Protease Inhibitor (Roche), Phospho STOP, 1 mM DTT). After centrifugation for 10 min at 4°C, supernatants were incubated immediately after with anti-FLAG (Sigma) at 4°C for 2 hr. Subsequently, the beads were washed twice with IP buffer, with IP buffer containing 1 M NaCl, with buffer F (20 mM Tris-HCl [pH 7.5], 1.2 mM EGTA, 250 mM sucrose, 150 mM NaCl, 1% Triton X-100, 0.5% NP-40), buffer F250 (buffer F, supplemented with 250 mM LiCl), with buffer D (20 mM HEPES-KOH [pH 7.9], 100 mM KCl, 0.2 mM EDTA, 5% glycerol, 0.5% NP-40, 0.2% Na-Deoxycholate), twice with buffer D400 (buffer D, supplemented with 400 mM KCl), and finally resuspended in buffer D. FLAG-UPF1 full-length protein affinity purification was performed as described above, and the protein was eluted with IP-buffer supplemented with 1 M NaCl. Recombinant UPF1, UPF2 and UPF3b purified from *E. coli* were a gift of Roberto Melero and Oscar Llorca (Madrid). They were diluted in buffer D and clarified with FLAG-M2 agarose or T7 agarose. The supernatant was subsequently incubated with FLAG-DHX34 beads, FLAG-KAT8, FLAG M2, T7-DHX34 (wild-type or deletion constructs), or T7 agarose beads and incubated for 2 hr at 4°C. For pull-down reactions in the presence of RNases, RNase A was added to 80 μg/ml. The beads were washed twice with buffer D, buffer D containing 400 mM KCl, 0.3 × buffer D, and once with buffer D. Proteins were eluted with 40 μl protein sample buffer and analyzed by SDS-PAGE and stained with Colloidal Coomassie (Life Technologies) and western blotting.

### In Situ UV Crosslinking mRNP Capture Assay

In situ UV crosslinking mRNP capture was performed as previously described (Piñol-Roma and Dreyfuss, 1992). HEK293T cells were transfected with 20 μg pcDNA3 × FLAG plasmids per 100 mm dishes. After 48 hr, RNA and protein complexes were UV crosslinked and scraped from 150 mm plates in 10 ml cold PBS, pelleted, and lysed for 10 min with 750 μl 10 mM Tris-HCl (pH 7.5), 60 mM NaCl, 5 mM MgCl_2_, 0.5 mM EDTA, 0.1 mM EGTA, 0.2% NP-40, 1 mM DTT, and minicomplete EDTA-free protease inhibitor (Roche Diagnostics). Cells were centrifuged, and supernatants were then denatured by the addition of an equal volume of 2 × binding buffer (20 mM Tris-HCl [pH 7.5], 1 M NaCl, 1% SDS, 0.2 mM EDTA). Approximately 50 μl packed bed volume of Oligo dT cellulose (Ambion) equilibrated in 1 × binding buffer was added to each fraction. The extracts were mixed with oligo dT cellulose overnight at room temperature on a rotating wheel and washed the next day three times with 1 ml of 1 × binding buffer. Captured mRNPs were eluted from the resin with 400 μl elution buffer (10 mM Tris-HCl [pH 7.5], 1 mM EDTA, minicomplete EDTA-free protease inhibitor) and 4 μl of RNase (Roche Diagnostics) for 30 min at 37°C. Liberated mRNA binding proteins were then precipitated by adding an equal volume of 20% TCA, incubating on ice for 20 min and pelleted in a refrigerated microcentrifuge for 20 min at 13,000 rpm. The precipitated proteins were then washed in ice-cold acetone and resuspended in 40 μl of SDS-PAGE sample buffer. Captured mRNA binding proteins were then resolved by 3%–8% Novex Tris-Acetate gels (Life Technologies) SDS-PAGE and analyzed by western blotting.

## Author Contributions

N.H. and J.F.C. conceived, designed, and interpreted the experiments. N.H. performed all the experiments and data analysis. J.F.C. supervised the whole project. The manuscript was cowritten by both authors.

## Supplemental Information

Document S1. Supplemental Experimental Procedures, Figures S1–S6, and Tables S1 and S2

Table S3. Microarray Gene Expression Analysis, Related to Figure S5

Document S2. Article plus Supplemental Information
