# Supplementary material for: The RNA Helicase DHX34 Activates NMD by Promoting a Transition from the Surveillance to the Decay-Inducing Complex
Source: Cell Rep. 2014 Sep 15;8(6):1845–56. doi: 10.1016/j.celrep.2014.08.020 (PMC4534575; doi:10.1016/j.celrep.2014.08.020)
Supplement: Document S1. Supplemental Experimental Procedures, Figures S1–S6, and Tables S1 and S2 [file mmc1.pdf]

Cell Reports, Volume *8*

Supplemental Information

The RNA Helicase DHX34 Activates NMD by Promoting a Transition from the Surveillance to the Decay-Inducing Complex

Nele Hug and Javier F. Cáceres

EXTENDED EXPERIMENTAL PROCEDURES

cDNA constructs

The plasmid pCGT7-DHX34 was created by inserting the DHX34 ORF (Ref Seq: NM_014681.3) between *Xba*I and *Bam*HI sites of the mammalian expression vector, pCGT7, which has been previously described (Cáceres et al., 1997). 3xFLAG-tagged expression vectors for UPF1 and DHX34 were generated by subcloning the 3xFLAG tag into the Multiple cloning site (MCS) of pcDNA3 (pCDNA 3xFLAG vectors) (Life Technologies). Full length DHX34 (RC213162) was obtained from Origene. The $DHX34^R$ shRNA resistant protein with silent mutations and the deletion mutants were cloned by PCR amplification, using the full length DHX34 as a template. Primer sequences are available upon request. Constructs used for the expression of UPF1 C126S and K498A mutant proteins were created in a similar way using full length UPF1 as template. The c-myc-UPF1 WT and G495R/G4957E were a generous gift from Lynne Maquat (University of Rochester). The SMG1 430 kDa clone (RC224277) (Ref Seq: NM_015092.3) clone was obtained from Origene. The pCIneoFLAG-eRF1, pCIneoFLAG-eRF3, pCIneoFLAG-UPF1 (wild-type and C126S, LECY181-184VRVD, VV204-205DI) and the deletion constructs (ΔCT, ΔNT, ΔCH) were a generous gift from Andreas Kulozik (University of Heidelberg). The FLAG-KAT8 expression construct was a generous gift from Wendy Bickmore (MRC HGU, Edinburgh).

Cell Culture

HEK293T cells were grown in high glucose Dulbecco's modified Eagle's medium (Life Technologies) supplemented with 10 % (v/v) fetal calf serum (Life

Technologies) and penicillin-streptomycin (Life Technologies) and incubated at 37°C in the presence of 5 % CO_2.

CLIP (UV Crosslinking and Immunoprecipitation)

UV-crosslinked cell pellets were lysed in CLIP lysis buffer (50 mM Tris-HCl, pH 7.4, 100 mM NaCl, 1 mM $MgCl_2$, 0.1 mM $CaCl_2$, 1 % NP-40, 0.5 % Na-Deoxycholate, 0.1 % SDS and Complete Mini tablets (Roche Diagnostics) supplemented with 7 M Urea. After sonication the lysate was diluted 7x in CLIP lysis buffer and treated with a low (0.05 U/µl) and a high RNase I dilution (2 U/µl) (Life Technologies) prior Immunoprecipitation with DHX34 antibody combined with Protein A-Dynabeads (Life Technologies). After washing 3 x with High salt buffer (50 mM Tris-HCl pH 7.4, 100 mM NaCl, 1 mM EDTA. 1 % NP-40, 0.5 % Na-Deoxycholate, 0.1 % SDS) and 2x with PNK buffer (20 mM Tris-HCl pH 7.4, 10 mM $MgCl_2$, 0.2 % Tween 20), the beads were subjected to CIP treatment and 3'RNA linker ligation and the RNA was labeled with [γ-32P] ATP, as previously described (Huppertz et al., 2014). Crosslinked RNA-protein complexes were separated on 3-8% Tris-Acetate Gels and transfered to Nitrocellulose membranes. Signals were detected with a phosphoimager.

Antibodies

Peptide-specific antibodies raised against human DHX34 were obtained from Eurogentec and purified according to standard procedures. The following antibodies were obtained commercially: UPF1 (A300-036A or A300-038A, Bethyl), UPF2 (sc-20227, Santa Cruz), UPF3b (sc-20231 or sc-48800, Santa Cruz) , XRN1 (A300-443A, Bethyl), XRN2 (A301-103A, Bethyl), PABP1 (4992, Cell Signaling), Phospho-(Ser/Thr) ATM/ATR Substrate Antibody (2851, Cell Signaling), Tubulin (TUB 2.1

clone, Sigma-Aldrich), SMG1 (ab30916, Abcam) or A301-535A, Bethyl), SMG7 (ab64847, Abcam), SMG6 (ab87539, Abcam), EIF4A3 (10463-1-AP, ProteinTech Group), CASC3 (A302-472A, Bethyl), DIS3 (14689-1-AP, ProteinTech Group), MOV10 (A500-009A, Bethyl), anti-FLAG (M2 clone, Sigma-Aldrich), anti-c-Myc (clone9E10, Sigma-Aldrich), eRF3 (ab49878, Abcam), monoclonal anti-T7 antibody (Novagen), CBP80 antibodies (A301-793A, Bethyl) and eIF3A (A320-002A-1, Bethyl). The Dcp1 Antibody was a generous gift from Bertrand Seraphin (IGBMC, Strasbourg), the SMG9 Antibody was provided by Shigeo Ohno (Yokohama City University) and the UPF3a antibody by Jens Lykke-Andersen (University of California-San Diego). For epitope-tag Immunopurifications, Anti-c-Myc Agarose (A7470, Sigma-Aldrich), Anti-FLAG M2 Affinity Gel (A2220, Sigma-Aldrich) and Anti-T7 Tag Antibody Agarose (69026, Novagen) were used. Protein G and protein A Sepharose were obtained from GE Healthcare. Secondary antibodies conjugated to Horse Radish Peroxidase and ChemiGlow detection reagent were obtained from BioRad and ProteinSimple, respectively.

ShRNA constructs

The target sequences used for DHX34 were CGACAAATCCAGCGGGAAC (shRNA I) and GAGCATCGACTGTACGAAA (shRNA). Target sequences for LUCIFERASE and UPF1, UPF2 and UPF3b have been previously described (Azzalin and Lingner, 2006; Metze et al., 2013; Wittmann et al., 2006). All target sequences were cloned into pSUPERpuro plasmids.

***In vitro* kinase assay**

HEK293T cells were transiently transfected with 20 µg of plasmids expressing FLAG-SMG1. Cells were lysed 48 h later through sonication in TGN buffer (50 mM Tris-HCl pH 7.5, 50 mM glycerophosphate, 150 mM NaCl, 10 % glycerol, 1 % Tween 20 supplemented with PhosphoSTOP and Complete Mini tablets (Roche Diagnostics) and 1 mM Dithiothreitol (DTT), as described (Canman et al., 1998). Following centrifugation at 13,000 g, 2 ml of extract were incubated with anti-FLAG beads for 2 h at 4ºC. Immunoprecipitates were washed twice with TGN buffer, once with 100 mM Tris (pH7.5) plus 0.5M LiCl, and twice with kinase buffer (10 mM HEPES (pH7.5), 50 mM glycerophosphate, 50 mM NaCl, 10 mM $MgCl_2$, 10 mM $MnCl_2$, 5 mM ATP, and 1 mM DTT). Kinase reactions were started by resuspending washed beads in kinase buffer containing 10 mCi [γ-32P] ATP and incubated with the purified proteins for the indicated time points at 30ºC. FLAG-DHX34 and FLAG-UPF1 proteins were immunopurified from HEK293T as described above and added to the kinase reactions. FLAG-UPF1 was used as substrate. Proteins were electrophoretically separated by SDS-PAGE and stained with Colloidal Coomassie (Life Technologies). Signals were detected and quantified with a phosphoimager and the AIDA software.

Microarray Analysis

For the Microarray analysis 4 µg of shRNA plasmids were transfected in six-well plates in three biological replicates; cells were expanded into 10 cm dishes and selected by growth in complete media supplemented with 0.75 µg/ml puromycin for 5 days post-transfection. Selective media was removed 12 h prior to harvesting. Total RNA was isolated using the Qiagen RNAeasy kit following manufacturer's

instruction. RNA quality was tested with the Agilent 2100 Bioanalyzer using RNA 6000 Nano Kit and NanoDrop 8000 spectrophotometer readings. For Microarray expression profiling, we used the Agilent 8 x 60 k arrays. 2 μg of total RNA were sent for further processing to Oxford Gene Technology, which provided the processed data. Microarray data was analyzed with R (www.r-project.org, version 3.0.0). Prior to quantile normalization, control probes, and probes with signals not significantly different from background (glsPosandSignif =0) were removed. Replicated probes on the array (identical Gene Names) were resolved by taking the average normalized signals. The R, Bioconductor package Limma (Smyth, 2004), was used to determine differentially expressed genes. To identify significant enrichment in the overlap for differentially expressed gene sets (adjusted p.value <0.05), we used a one tailed Fisher's exact test (Figure S5C). Pearson's R and associated significance for data used in Figure S5B (genes with Log2 FC differential expression >1 and adjusted p.value <0.05) were calculated using the R function cor.test . RT-qPCR to validate microarray results was performed as described (Longman et al., 2013).

SUPPLEMENTAL REFERENCES

Azzalin, C.M., and Lingner, J. (2006). The human RNA surveillance factor UPF1 is required for S phase progression and genome stability. Curr. Biol. *16*, 433–439.

Cáceres, J.F., Misteli, T., Screaton, G.R., Spector, D.L., and Krainer, A.R. (1997). Role of the modular domains of SR proteins in subnuclear localization and alternative splicing specificity. J. Cell Biol. *138*, 225–238.

Canman, C.E., Lim, D.S., Cimprich, K.A., Taya, Y., Tamai, K., Sakaguchi, K., Appella, E., Kastan, M.B., and Siliciano, J.D. (1998). Activation of the ATM kinase by ionizing radiation and phosphorylation of p53. Science *281*, 1677–1679.

Huppertz, I., Attig, J., D'Ambrogio, A., Easton, L.E., Sibley, C.R., Sugimoto, Y., Tajnik, M., König, J., and Ule, J. (2014). iCLIP: protein-RNA interactions at nucleotide resolution. Methods *65*, 274–287.

Longman, D., Hug, N., Keith, M., Anastasaki, C., Patton, E.E., Grimes, G., and Cáceres, J.F. (2013). DHX34 and NBAS form part of an autoregulatory NMD circuit that regulates endogenous RNA targets in human cells, zebrafish and Caenorhabditis elegans. Nucleic Acids Res. *41*, 8319–8331.

Metze, S., Herzog, V.A., Ruepp, M.-D., and Mühlemann, O. (2013). Comparison of EJC-enhanced and EJC-independent NMD in human cells reveals two partially redundant degradation pathways. RNA *19*, 1432–1448.

Smyth, G.K. (2004). Linear models and empirical bayes methods for assessing differential expression in microarray experiments. Stat. Appl. Genet. Mol. Biol. *3*, Article3.

Wittmann, J., Hol, E.M., and Jäck, H.-M. (2006). hUPF2 silencing identifies physiologic substrates of mammalian nonsense-mediated mRNA decay. Mol. Cell. Biol. *26*, 1272–1287.

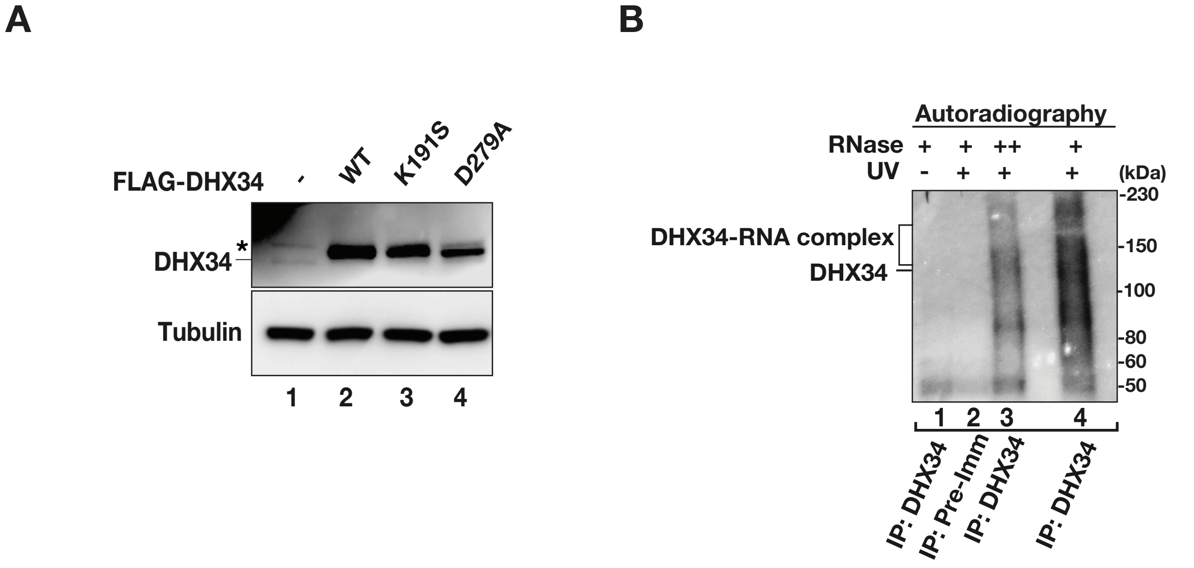

Figure S1, Related to Figure 1. (A) Western Blot showing the expression levels of FLAG-DHX34 (wild-type and point mutations) used in the Oligo-dT purification assay by probing with a DHX34 specific antibody. The lower band corresponds to DHX34, whereas an asterisk above DHX34 indicates an unspecific band. (B) Analysis of UV-crosslinked endogenous DHX34-RNA complexes. HEK293T cells were UV-crosslinked, followed by immunoprecipitation of endogenous DHX34 protein with a specific antibody in the presence of high (++) or low (+) RNase concentrations. The crosslinked RNA was radiolabeled for detection. IPs were separated by SDS-PAGE, transferred to nitrocellulose membrane and exposed to a phosphoimager.

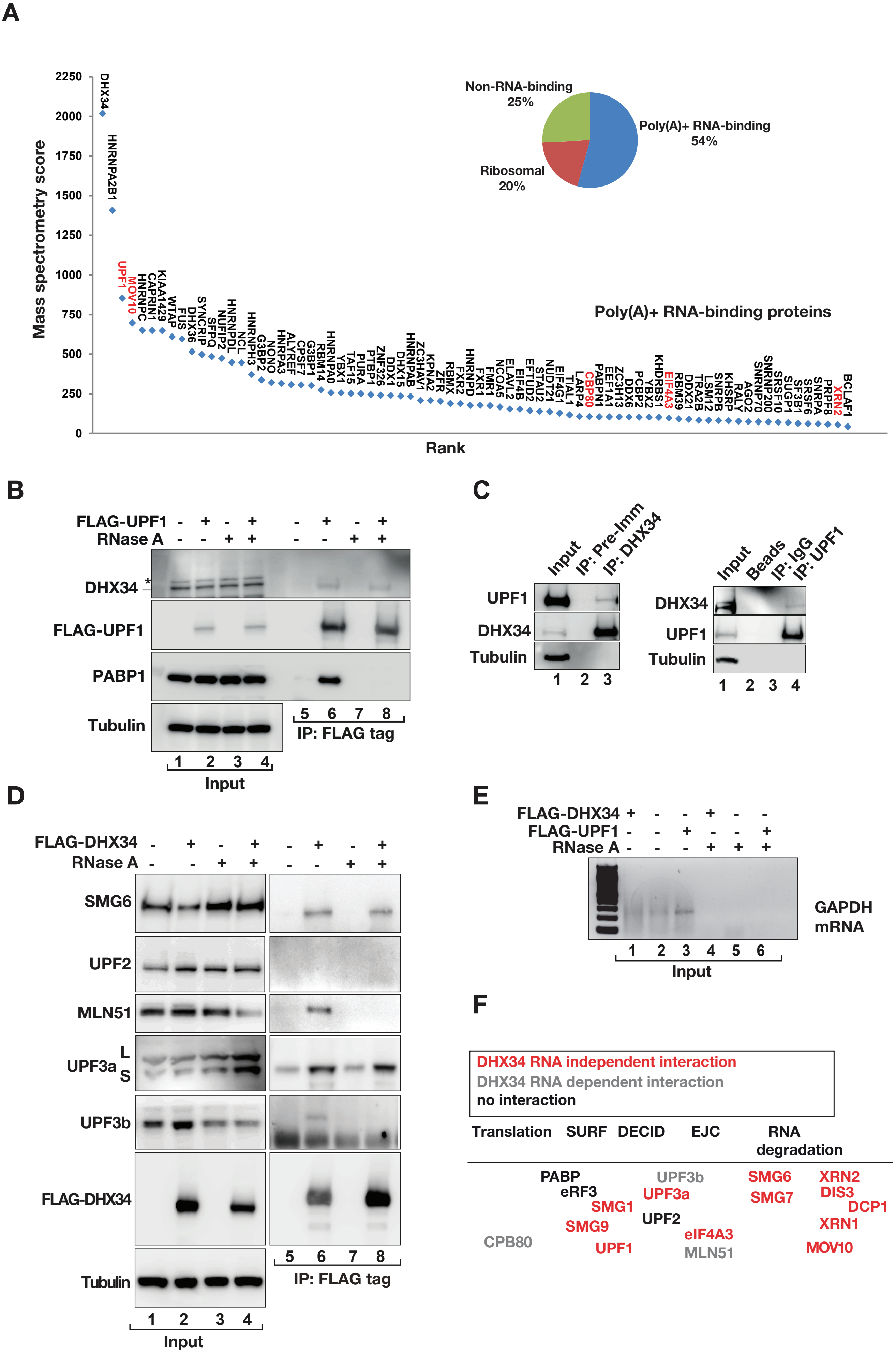
A
Mass spectrometry score
2250
2000
1750
1500
1250
1000
750
500
250
0
Rank
Poly(A)+ RNA-binding proteins
Non-RNA-binding 25%
Poly(A)+ RNA-binding 54%
Ribosomal 20%
DHX34
HNRNPA2B1
UPF1
MOV10
HNRNPC
CAPRIN1
KIAA1429
WTAP
FUS
DHX36
SYNCRIP
SFPQ
NUFIP2
HNRNPDL
NCL
HNRNPH3
G3BP2
NONO
HNRPA3
ALYREF
CPSF7
G3BP1
RBM14
HNRNPA0
YBX1
TAF15
PURA
PTBP1
ZNF326
DDX1
DHX15
HNRNPAB
ZC3HAV1
KPNA2
ZFR
RBMX
FXR2
HNRNPD
FXR1
FMR1
NCOA5
ELAVL2
EIF4B
EFTUD2
STAU2
NUDT21
EIF4G1
TIAL1
LARP4
CBP80
PABPN1
EEF1A1
PABPC1
ZC3H13
DDX6
PCBP2
YBX2
KHDRBS1
EIF4A3
RBM39
DDX21
TRA2B
LSM12
SNRPB
KHSRP
RALY
AGO2
SNRNP70
SNRNP200
SRSF10
SUGP1
SF3B1
SRSF6
SNRPA
PRPF8
XRN2
BCLAF1
B
FLAG-UPF1 - + - + - + - +
RNase A - - + + - - + +
DHX34 *
FLAG-UPF1
PABP1
Tubulin
1 2 3 4
Input
5 6 7 8
IP: FLAG tag
C
Input
IP: Pre-Imm
IP: DHX34
UPF1
DHX34
Tubulin
1 2 3
Input
Beads
IP: IgG
IP: UPF1
DHX34
UPF1
Tubulin
1 2 3 4
D
FLAG-DHX34 - + - + - + - +
RNase A - - + + - - + +
SMG6
UPF2
MLN51
UPF3a L S
UPF3b
FLAG-DHX34
Tubulin
1 2 3 4
Input
5 6 7 8
IP: FLAG tag
E
FLAG-DHX34 + - - + - -
FLAG-UPF1 - - + - - +
RNase A - - - + + +
GAPDH mRNA
1 2 3 4 5 6
Input
F
DHX34 RNA independent interaction
DHX34 RNA dependent interaction
no interaction
Translation
SURF
DECID
EJC
RNA degradation
PABP
eRF3
SMG1
SMG9
UPF1
CPB80
UPF3b
UPF3a
UPF2
eIF4A3
MLN51
SMG6
SMG7
XRN2
DIS3
DCP1
XRN1
MOV10

Figure S2, Related to Figure 2. (A) Endogenous DHX34 protein was immunoprecipitated from HEK293T cells with a specific antibody or the pre-immune serum and analyzed by mass spectrometry. A list of interacting poly(A)+ RNA-binding proteins were ranked accordingly to the score detected in the mass spectrometry analysis. Proteins involved in NMD are marked in red and were validated in Figure 2A. The pie chart indicates the proportion of poly (A)+ RNA-binding proteins among the interactors. (B) HEK293T cells were transiently transfected with FLAG-UPF1. Immunoprecipitation was performed as in Figure 2 and RNase A was added to half of each extract before immunoprecipitation. Inputs (0.5%) and anti-FLAG-IPs (20%) were probed for the indicated proteins. The lower band corresponds to DHX34. The asterik above DHX34 indicates an unspecific band. (C) Co-precipitation of endogenous UPF1 and DHX34 proteins. HEK293T cell extracts were immunoprecipitated with DHX34 or UPF1-specific antibodies, respectively and probed with the corresponding antibodies in Western blot assays. For anti-DHX34 IPs the pre-immune serum (Pre_Imm) served as a negative control. For anti-UPF1-IPs no antibody (Beads) or goat IgGs (IgG) served as a negative control. (D) Immunoprecipitation of transiently transfected FLAG-DHX34 from HEK293T cells in the absence or presence of RNase A. Inputs (0.5%) and anti-FLAG-IPs (20%) were probed with the indicated antibodies. UPF3a L and S represent two different isoforms. (E) A control of RNase A activity. Cellular GAPDH mRNA was analyzed using semiquantitative RT-PCR to confirm the removal of cellular RNAs upon incubation with RNase A. (F) Table summarizing DHX34 interactors involved at different steps of the NMD pathway.

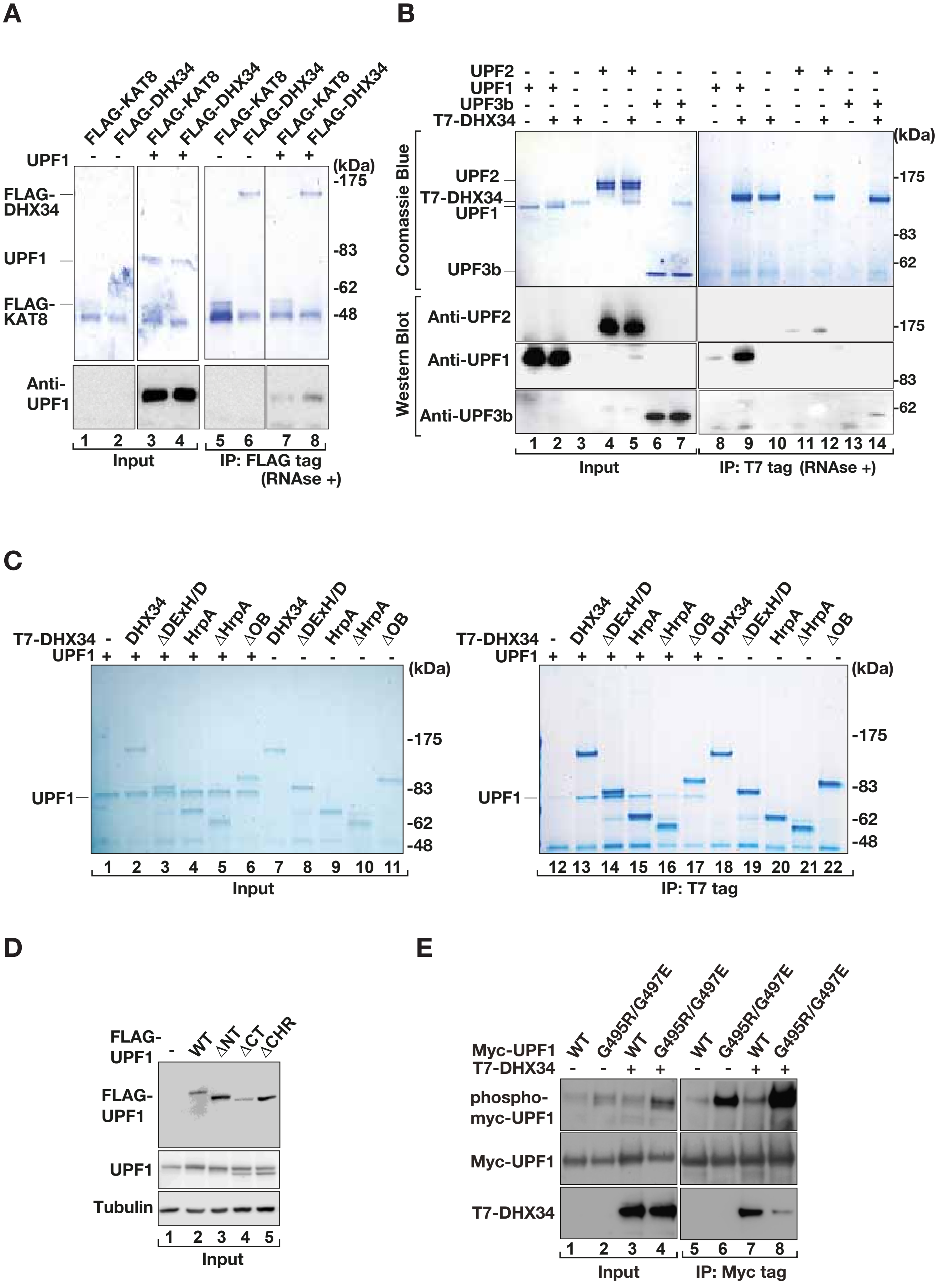
A
FLAG-KAT8
FLAG-DHX34
UPF1
(kDa)
-175
-83
-62
-48
FLAG-DHX34
UPF1
FLAG-KAT8
Anti-UPF1
1 2 3 4 5 6 7 8
Input
IP: FLAG tag (RNAse +)
B
UPF2
UPF1
UPF3b
T7-DHX34
Coomassie Blue
Western Blot
Anti-UPF2
Anti-UPF1
Anti-UPF3b
1 2 3 4 5 6 7 8 9 10 11 12 13 14
Input
IP: T7 tag (RNAse +)
C
T7-DHX34
UPF1
DHX34
ΔDExH/D
HrpA
ΔHrpA
ΔOB
Input
IP: T7 tag
D
FLAG-UPF1
WT
ΔNT
ΔCT
ΔCHR
UPF1
Tubulin
1 2 3 4 5
Input
E
Myc-UPF1
T7-DHX34
WT
G495R/G497E
phospho-myc-UPF1
Myc-UPF1
T7-DHX34
1 2 3 4 5 6 7 8
Input
IP: Myc tag

Figure S3, Related to Figure 3. (A) Interaction between purified FLAG-DHX34 and recombinant UPF1 proteins, which were mixed in a 1:1 ratio and pulled down using anti-FLAG beads in presence of RNase A. Purified FLAG-KAT8 served as a negative control. FLAG-DHX34 was purified from HEK293T cells under high stringency conditions that eliminated detectable endogenous UPF1 from the Immunopurifications. Inputs (5%) and pulldown fractions (20%) were analyzed by Coomassie Staining or Western blotting with the indicated antibodies. (B) Interaction between purified T7-DHX34 and purified FLAG-UPF1, and with recombinant UPF2 and UPF3b proteins, which were mixed in a 1:1 ratio and pulled down using T7 beads in presence of RNase A. T7-DHX34 and FLAG-UPF1 were purified from HEK293T cells under stringent conditions, and analyzed as indicated above. Since full length UPF1 and DHX34 are similar in size no distinct bands can be detected in the pulldown fractions by Coomassie Blue staining. (C) Interaction between purified T7-DHX34 (wild-type and deletion mutants) and recombinant UPF1 proteins, which were mixed in a 1:1 ratio and pulled down using anti-T7 beads. Inputs (5%) and pulldown fractions (20%) were analyzed after SDS-PAGE by Coomassie Staining. (D) HEK293T cells were transiently transfected with FLAG-UPF1 (wild-type and deletions) under the same experimental conditions as in Figure 3C. To detect the level of overexpression of FLAG-UPF1 Inputs were probed with anti-UPF1 antibodies. (E) Myc-UPF1 (wild-type) or a ATPase deficient mutant (G495R/G497E), which accumulates in a hyperphosphorylated form were co-expressed with T7-DHX34 in HEK293T cells. Inputs (0.5%) and anti-Myc-IPs (20%) were probed with the indicated antibodies.

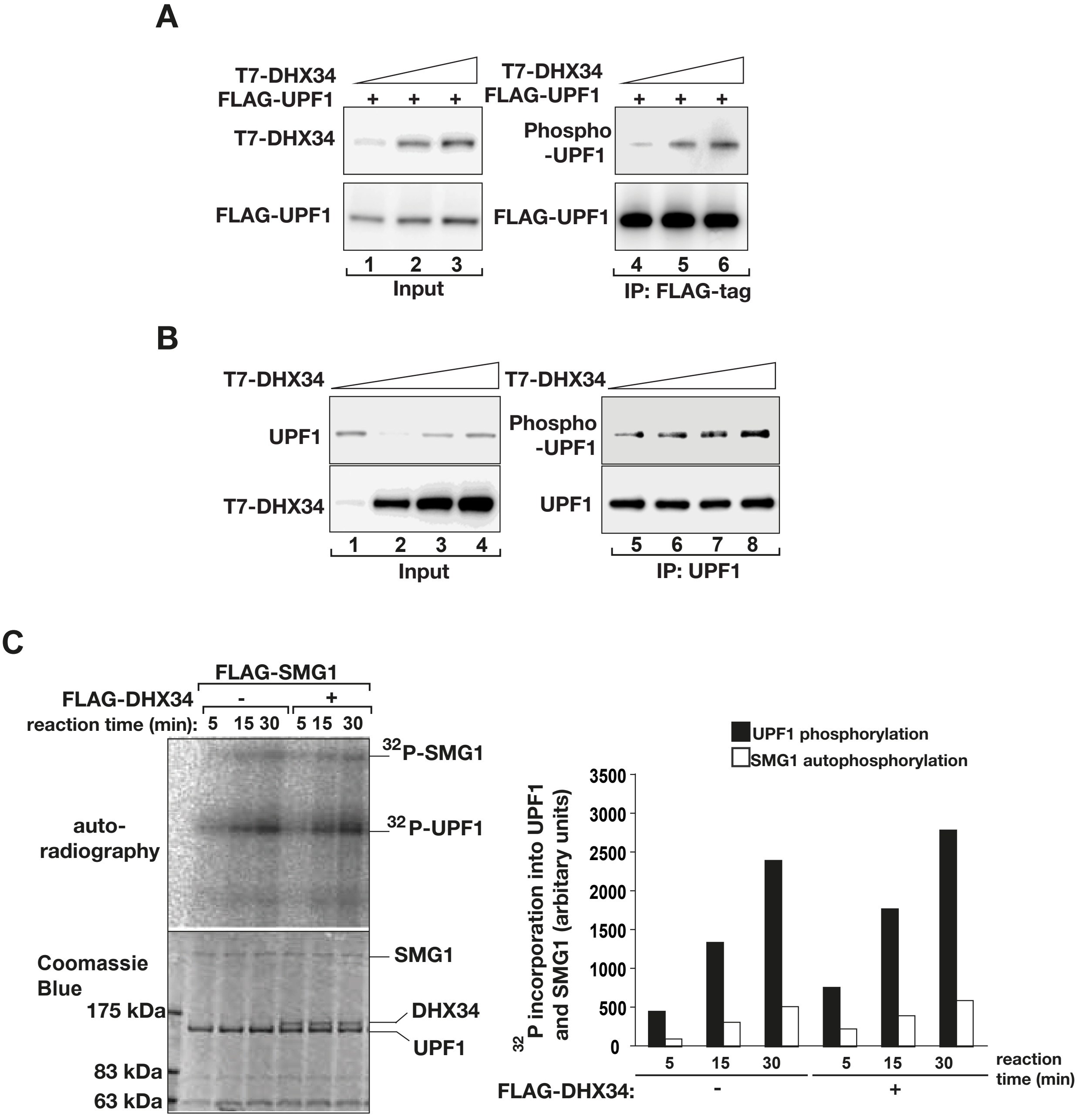

Figure S4, Related to Figure 4. (A) Cells were co-transfected with 2 µg FLAG-UPF1 expression vector and increasing amounts of T7-DHX34 (2 µg, 4 µg and 8 µg). Inputs (0.5%) and anti-FLAG-IPs (20%) were probed with the indicated antibodies. For detection of UPF1 phosphorylation (phospho-UPF1), anti-FLAG-IPs were probed with a phospho-(Ser/Thr) ATM/ATR substrate antibody. (B) Cells were transfected with increasing amounts of T7-DHX34 (0.5 µg, 2 µg, 4 µg and 8 µg). Inputs (0.5%) and anti-UPF1-IPs (20%) were probed with the indicated antibodies. For detection of UPF1 phosphorylation (phospho-UPF1), the anti-UPF1-IPs were probed with a phospho-(Ser/Thr) ATM/ATR substrate antibody. (C) *In vitro* kinase assays with anti-FLAG-SMG1 immunoprecipitates as the kinase source were performed in presence of purified FLAG-DHX34 and empty vector sample (-) using FLAG-UPF1 as the sub-strate. Kinase reactions were separated by SDS-PAGE, stained with Coomassie Blue and exposed to a phosphoimager. Quantitation of the phosphorylation level of SMG1 kinase and UPF1 *in vitro* are shown in the right panel.

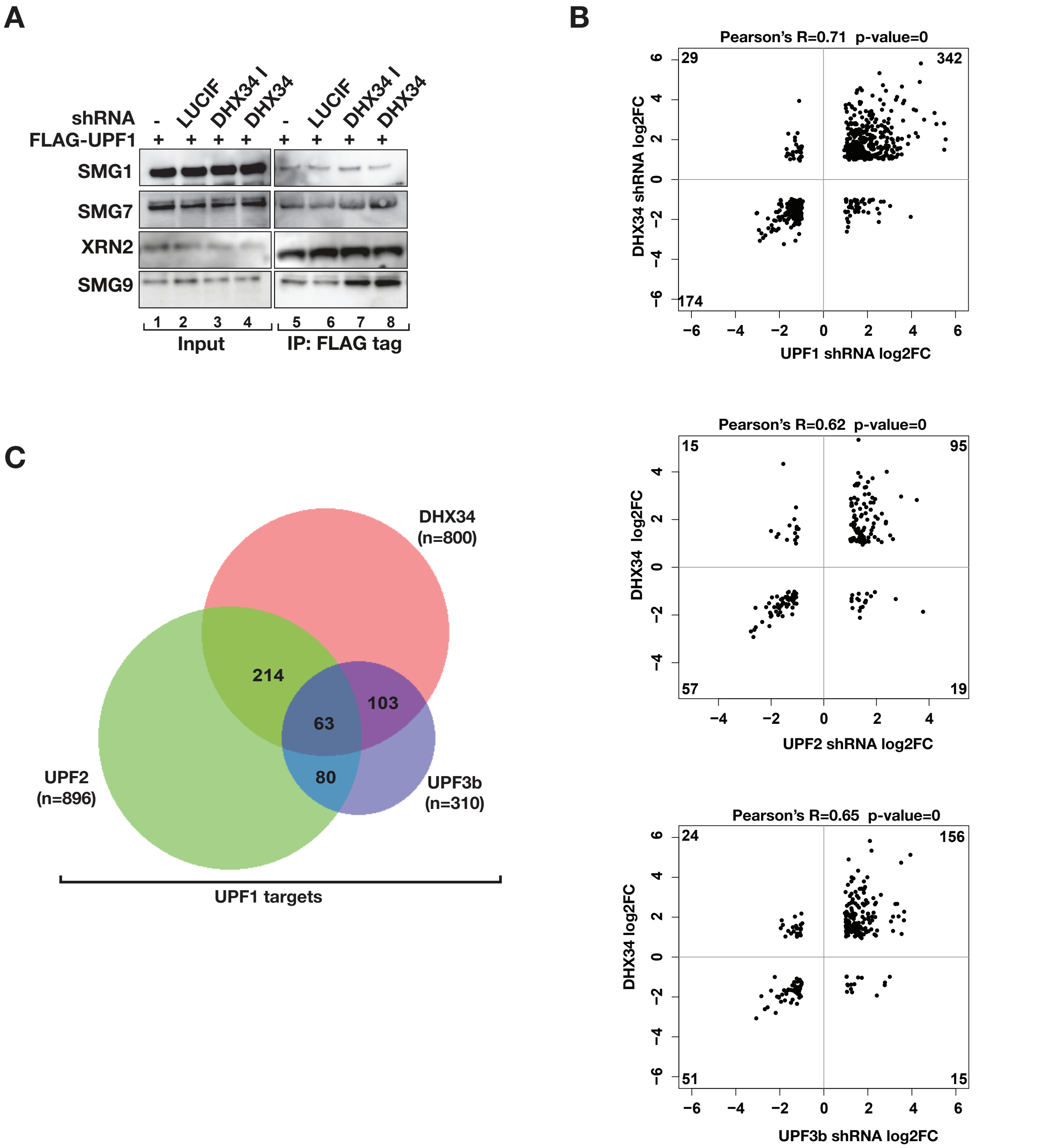

Figure S5, Related to Figure 5. (A) Depletion of DHX34 does not disrupt the interaction of FLAG-UPF1 with SMG1, SMG7, XRN2 or SMG9. Input (0.5%) and anti-FLAG-IPs (20%) were probed with the indicated antibodies. (B) Microarray analysis of DHX34, UPF1, UPF2 and UPF3b. DHX34 co-regulates RNA targets with all three core NMD factors UPF1, UPF2 and UPF3b. Scatter plots show a significant positive correlation for the regulation of target genes that significantly changed by more than 2 fold in DHX34, UPF1, UPF2 and UPF3b samples. Pearson's R and p-values are indicated. Validation of 13 random genes and the depletion by RT-qPCR are shown in Table S2. The overall effects of the individual depletions on gene expression can be found in Table S3. (C) The Venn Diagram illustrates the overlap for RNA targets between DHX34, UPF2 and UPF3b depleted samples found in UPF1 depleted samples. Only targets that were upregulated significantly and more than 1.5 fold are included. Areas are proportional to the number of genes regulated. For each overlap, the target co-regulation is significant ($p < 2.2e-16$).

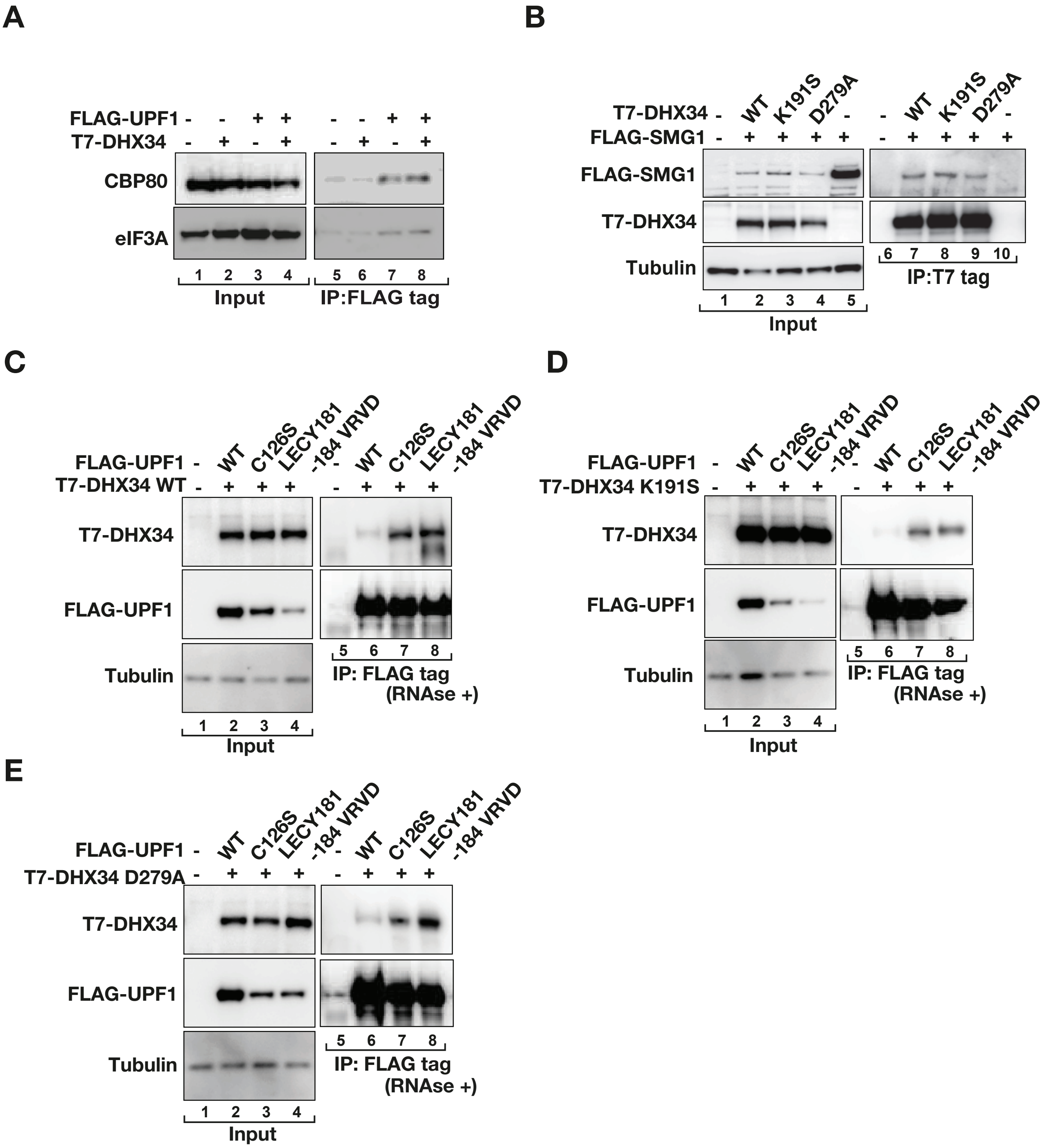

Figure S6, Related to Figure 6. (A) Overexpression of DHX34 does not disrupt the interaction of UPF1 with CBP80 or eIF3A. Inputs (0.5%) and anti-FLAG-IPs (20%) from HEK293T cells co-expressing FLAG-UPF1 and T7-DHX34 were probed for the indicated proteins. (B) Interaction of T7-DHX34 (wild-type or the ATPase deficient mutants K191S or D279A) with FLAG-SMG1. Inputs (0.5%) and anti-T7-IPs (20%) from cells co-transfected with T7-DHX34 and FLAG-SMG1 were probed for the indicated proteins. (C), (D) and (E) Mutation of the DHX34 helicase domain does not affect the preferential binding to the SURF complex. Inputs (0.5%) and anti-FLAG-IPs (20%) from cells co-expressing FLAG-UPF1 (wild-type, C126S or LECY181-184VRVD mutants) and T7-DHX34 (wild-type (C), K191S (D) and D279A (E) mutants) were probed for the indicated proteins.

Hug and Caceres, Table S1: DHX34 interactors

Gene name	Mass spec. Score	% Coverage	Proteins	Unique Peptides	Peptides
**Splicing and RNA processing**					
HNRNPA2B1	1407.46	59.21	1	16	36
UPF1	853.45	35.06	2	28	30
HNRNPC	651.06	40.08	7	12	17
WTAP	609.06	29.55	1	10	14
FUS	595.76	28.93	2	10	19
DHX36	515.9	15.63	4	12	13
SYNCRIP	497.66	22.28	2	7	13
SFPQ	484.74	24.05	1	14	15
HNRNPDL	448.13	20.48	1	2	14
NCL	445.9	25.54	2	12	14
CPSF7	306.66	28.88	4	9	11
HNRNPA0	256.74	18.36	1	5	8
YBX1	256.37	39.2	1	5	8
PTBP1	244.65	18.64	3	7	8
DDX1	240.12	10.54	1	4	6
DHX15	236.3	7.78	2	5	6
HNRNPAB	233.74	22.86	5	4	6
RBMX	189.31	15.6	1	7	7
HNRNPD	177.59	34.82	9	2	5
STAU2	139.69	7.25	3	4	4
NUDT21	137.96	28.63	1	6	6
TIAL1	117.39	7.94	4	3	3
CBP80	106.92	5.32	1	3	3
PABPN1	104.49	9.12	2	2	2
DDX6	103.88	12.01	1	4	4
PCBP2	103.19	25.62	12	3	3
YBX2	102.84	21.93	2	2	4
EIF4A3	97.29	7.79	1	2	2
RBM39	91.81	15.73	6	3	3
DDX21	89.43	4.62	2	2	3
TRA2B	83.38	13.3	3	3	3
SNRPB	81.96	3.9	7	2	2
KHSRP	80.14	2.96	2	2	2
RALY	77.11	20.85	1	2	2
SNRNP70	73.57	6.07	2	3	2
SNRNP200	73.08	1.03	1	2	2
SRSF10	71.36	40.58	8	2	3
SUGP1	68.13	5.44	3	2	2
SF3B1	64.2	8.71	2	2	2
SRSF6	61.06	6.45	3	2	2
SNRPA	60.98	14.18	1	2	2
PRPF8	58.68	0.9	1	2	2
XRN2	55.22	4.12	3	2	2

**Transcription & Translation**					
MOV10	697.47	21.24	1	18	19
CAPRIN1	649.48	26.8	2	14	21
KIAA1429	648.76	12.47	1	16	20
NONO	320.74	29.32	2	9	18
HNRPA3	317.08	19.2	3	4	10
RBM14	274.9	11.81	1	7	7
TAF15	253.9	28.52	2	7	11
PURA	251.24	18.01	1	3	5
ZNF326	241.66	12.54	1	6	7
FXR2	188.84	5.2	1	1	5
FXR1	177.57	9.65	3	2	6
FMR1	175.45	9.45	10	4	6
NCOA5	167.59	18.31	1	5	6
ELAVL2	154.52	13.29	3	3	4
EIF4B	153.7	33.51	7	2	4
EFTUD2	143.95	7.27	4	3	3
EIF4G1	128.33	3.21	6	4	4
EEF1A1	104.18	6.35	4	3	3
KHDRBS1	101.49	7.42	3	4	4
AGO2	75.15	2.79	2	2	4
BCLAF1	43.4	2.34	7	2	2
**tRNA**					
RTCB	137.02	8.71	1	3	4
**Ribosomal proteins**					
RPL18	258.01	30.19	5	4	5
RPS3A	257.56	31.71	3	6	7
RPS8	230.3	20.74	2	3	5
RPL12	188.31	33.33	1	4	6
RPL19	187.88	16.84	1	3	4
RPL4	164.79	18.84	2	5	5
RPL7A	160.18	25.13	2	4	4
RPS7	120.58	22.99	2	3	4
RPL7	98.1	14.9	2	3	4
RPL3	113.12	13.77	3	3	4
RPS14	112.41	24.5	1	4	3
RPL13	101.11	15.17	1	3	2
RPLP0	98.41	16.34	5	2	3
RPS9	93.87	14.43	1	3	3
RPL23A	92.9	10.9	5	2	2
RPL13A	88.67	13.79	1	3	3
RPL8	85.18	12.16	5	2	2
RPS2	80.05	8.53	1	3	3
RPL26L1	77.66	14.06	4	2	2
RPS4X	74.83	7.98	1	2	2
RPS13	70.95	19.87	1	3	3
RPL6	67.05	14.24	2	4	4
RPS26	66.42	20.87	1	2	2
RPS11	60.17	9.49	2	2	2
RPL27	60.15	12.5	1	2	2
RPS24	55.72	15.38	5	2	2
RPL32	50.25	12.78	3	2	2

**Nuclear transport**					
ALYREF	308.47	27.63	2	3	5
KPNA2	207.92	11.72	1	4	4
**Protein transport**					
SEC16A	123.6	4.36	5	5	5
**Cell Cycle**					
CDK11A	126.51	9.57	19	4	4
**DNA replication and repair**					
XRCC6	274.59	15.27	1	7	8
XRCC5	148.14	11.2	1	5	5
DDB1	86.15	5.57	2	2	2
MCM7	66.86	7.97	5	2	2
**Signal transduction**					
G3BP2	338.19	17.92	3	6	9
G3BP1	302.71	31.33	1	7	8
**Cell Metabolism**					
ATP5B	623.25	34.4	1	12	19
PRDX4	365.38	45.02	1	11	15
ATP5A1	343.98	15.9	3	8	10
PHGDH	133.05	9.02	2	4	4
SLC25A6	93.85	13.29	2	2	2
PKM2	91.48	4.59	6	2	2
FASN	84.34	1.91	1	3	3
LDHA	78.95	26.32	8	2	2
**Chaperones**					
HSPA1A	869.67	32.29	2	15	24
TCP1	206.28	13.85	1	4	6
HSP90AA1	123.19	4.92	2	1	3
HSP90AB1	117.5	4.42	1	1	3
BAG4	57.27	5.7	2	2	2
**Cytoskeleton**					
DSP	653.37	7.35	1	15	18
TUBA1B	621.81	28.16	1	9	15
TUBB	587.16	45.75	3	4	18
ACTA1	134.15	9	19	2	3
**Histones**					
HIST1H2AH	66.6	21.88	10	2	2
**Proteolysis**					
UBA1	131.51	6.05	1	3	4
KLHL12	68.71	7.39	2	2	2
DCD	57.05	18.18	2	2	2
CBLL1	112.07	6.53	2	3	4
**Viral response**					
ZC3HAV1	208.8	9.49	3	3	3
**Uncharacterized**					
NUFIP2	473.58	23.31	1	11	13
ZFR	203.51	4.26	2	3	3
LARP4	108.22	9.6	6	3	4
ZC3H13	104.13	2.94	2	4	4
LSM12	83.2	38.94	3	3	3
CCDC51	59.52	10.26	2	2	2

**Others**					
PGAM5	85.33	6.67	3	2	2
ALB	88.94	4.85	5	2	3
IGHG2	42.48	3.6	2	2	2

Hug and Caceres, Table S2: Validation of Microarray analysis with RT qPCR

	control		DHX34 shRNA			UPF1 shRNA			UPF2 shRNA			UPF3B shRNA		
gene	qPCR	SD	qPCR	SD	array	qPCR	SD	array	qPCR	SD	array	qPCR	SD	array
PBXIP1	1	0.14	8.82	0.18	4.82	3.95	0.58	2.21	2.63	0.25	1.49	3.43	0.29	2.04
GCNT3	1	0.17	5.37	0.33	4.02	2.60	0.30	1.61	1.29	0.10	0.93	1.08	0.06	1.08
ALDH3B1	1	0.08	1.25	0.08	1.15	2.19	0.61	2.29	1.03	0.18	0.91	1.92	0.12	1.54
ZDHHC11	1	0.13	2.30	0.18	1.95	3.47	0.10	3.52	1.23	0.07	1.18	0.49	0.13	0.58
INHBA	1	0.14	2.68	0.16	1.95	1.71	0.34	1.00	1.51	0.38	0.98	1.38	0.29	0.83
CPEB4	1	0.05	5.48	0.95	3.40	0.80	0.48	1.16	0.76	0.07	1.09	1.42	0.59	1.62
FOXP2	1	0.07	1.59	0.18	1.22	1.12	0.46	0.73	0.44	0.07	0.41	1.00	0.11	0.56
MGP	1	0.16	0.67	0.14	0.66	1.83	0.06	1.39	2.45	0.22	1.57	2.66	0.17	2.28
SH3TC1	1	0.11	2.11	0.15	1.74	1.20	0.13	1.65	0.72	0.07	1.00	0.84	0.04	1.29
EGF	1	0.13	1.34	0.26	1.22	9.34	2.01	5.02	5.03	0.68	1.58	4.03	0.09	1.09
DTNB	1	0.06	0.94	0.10	0.53	2.24	0.69	1.38	1.71	0.10	1.03	0.97	0.08	0.95
IL6R	1	0.12	2.88	0.44	2.40	1.83	0.14	1.16	1.97	0.06	0.93	2.82	0.41	1.48
BMP2	1	0.13	4.55	0.82	2.03	2.10	0.10	1.06	1.26	0.06	1.39	3.57	0.83	2.68
DHX34	1	0.17	0.49	0.07	0.37	0.97	0.20	1.41	0.89	0.19	0.86	0.79	0.04	0.97
UPF1	1	0.15	0.98	0.09	0.82	0.13	0.07	0.26	1.11	0.12	2.31	1.02	0.12	1.36
UPF2	1	0.07	1.19	0.17	1.16	0.97	0.12	1.23	0.39	0.16	0.21	0.83	0.16	1.01
UPF3B	1	0.15	1.04	0.27	0.50	0.73	0.22	1.14	1.26	0.18	1.42	0.22	0.27	0.21
